# A Composite Hydrogel Functionalized by Borosilicate Bioactive Glasses and VEGF for Critical‐Size Bone Regeneration

**DOI:** 10.1002/advs.202400349

**Published:** 2024-05-07

**Authors:** Chao Huang, Shun Shi, Muyan Qin, Xiao Rong, Zichuan Ding, Xiaoxue Fu, Weinan Zeng, Lei Luo, Deping Wang, Zeyu Luo, Yiwen Li, Zongke Zhou

**Affiliations:** ^1^ Department of Orthopaedics West China Hospital Sichuan University Chengdu Sichuan 610041 P. R. China; ^2^ College of Polymer Science and Engineering State Key Laboratory of Polymer Materials Engineering Sichuan University Chengdu Sichuan 610065 P. R. China; ^3^ School of Materials Science and Engineering Tongji University Shanghai 201804 P. R. China; ^4^ Department of Ultrasound West China Hospital Sichuan University Chengdu Sichuan 610041 P. R. China; ^5^ West China School of Clinical Medicine Sichuan University Chengdu Sichuan 610041 P. R. China

**Keywords:** angiogenesis, bioactive glass, bone regeneration, hydrogel, osteogenic differentiation, VEGF

## Abstract

Critical‐size bone defects pose a formidable challenge in clinical treatment, prompting extensive research efforts to address this problem. In this study, an inorganic–organic multifunctional composite hydrogel denoted as PLG‐*g*‐TA/VEGF/Sr‐BGNPs is developed, engineered for the synergistic management of bone defects. The composite hydrogel demonstrated the capacity for mineralization, hydroxyapatite formation, and gradual release of essential functional ions and vascular endothelial growth factor (VEGF) and also maintained an alkaline microenvironment. The composite hydrogel promoted the proliferation and osteogenic differentiation of rat bone marrow mesenchymal stem cells (rBMSCs), as indicated by increased expression of osteogenesis‐related genes and proteins in vitro. Moreover, the composite hydrogel significantly enhanced the tube‐forming capability of human umbilical vein endothelial cells (HUVECs) and effectively inhibited the process of osteoblastic differentiation of nuclear factor kappa‐B ligand (RANKL)‐induced Raw264.7 cells and osteoclast bone resorption. After the implantation of the composite hydrogel into rat cranial bone defects, the expression of osteogenic and angiogenic biomarkers increased, substantiating its efficacy in promoting bone defect repair in vivo. The commendable attributes of the multifunctional composite hydrogel underscore its pivotal role in expediting hydrogel‐associated bone growth and repairing critical bone defects, positioning it as a promising adjuvant therapy candidate for large‐segment bone defects.

## Graphical abstract

The PLG‐g‐TA/VEGF/Sr‐BGNPs hydrogel synergistically accelerated the in‐situ regeneration of bone tissue by promoting the proliferation, migration, and osteogenic differentiation of BMSCs; promoting the differentiation of HUVECs to form blood vessels; and inhibiting the differentiation of osteoclasts and their bone resorption ability. This study proposes a new way of treating bone defects in the future, and PLG‐g‐TA/VEGF/Sr‐BGNPs hydrogel is a promising biomaterial for artificial renewable bone grafts.

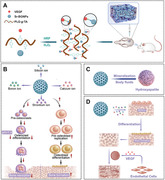

## 1 Introduction

Bone defects are typically observed in patients with severe trauma, infection, or surgical resection of bone tumors. Although bone has a certain self‐healing ability, surgical intervention is required when the defect exceeds the body's self‐healing abilities.^[^ ^1^ ^]^ Autologous bone grafting is the “gold standard” for treating bone defects. However, there are still some problems, such as insufficient donor sources, pain in the donor area, nerve damage, and recurrent fractures, which have limited its application.^[^ ^2^ ^]^ Allograft bone grafting is another option for treating bone defects. However, this approach is limited due to concerns such as potential disease transmission and social ethics risks.^[^ ^3^ ^]^ The development of artificial regenerative bone graft biomaterials to address the needs of clinical applications has become a popular research topic in biomedical regeneration. Hydrogel is formed by water‐soluble macromolecules via cross‐linking with a 3D network structure, which can mimic the natural extracellular matrix (ECM) structure with an interpenetrating porous structure.^[^ ^4^ ^]^ The high hydrophilicity of hydrogels allows them to store biological proteins, growth factors, and cells.^[^ ^5^ ^]^ Hydrogels are also easily modifiable and have tunable physical, chemical, and biological properties.^[^ ^6^ ^]^ Thus, hydrogels are potential candidates for tissue substitutes and have been widely used in many regenerative medicine applications.^[^ ^7^ ^]^ However, there are some deficiencies that cause hydrogels to fail to meet the requirements of artificial regenerative bone graft biomaterials, such as low mechanical stiffness and poor ability to induce mineralization.^[^ ^8^ ^]^ The construction of bioactive inorganic‒organic composite hydrogels is an effective strategy to enhance the mechanical strength of hydrogels and to endow them with new properties as artificial regenerative bone graft biomaterials.^[^ ^9^ ^]^ These composite hydrogels can create an environment that closely mimics the ECM, facilitating cell‐matrix interactions and signaling pathways essential for osteogenesis.^[^ ^10^ ^]^

Osteonectin, a protein found in bone and dentin, is associated with osteogenesis. It consists of 285–287 amino acid residues, and its molecular structure can be divided into four domains, among which amino‐terminal structural domain I contains glutamic acid‐rich sequences, and hydroxyapatite (HA)‐binding activity is thought to occur in domain I.^[^ ^11^ ^]^ Therefore, the present study focused on glutamic acid‐based hydrogels. However, few studies have reported the single use of poly(amino acid)‐based hydrogels in bone tissue engineering (BTE) because the inherent poor mechanical and weak osteogenic properties of hydrogels limit their application in the repair of load‐bearing bone defects.^[^ ^12^ ^]^ The addition of inorganic compounds to inorganic‒organic composite hydrogels is an effective method for promoting the application of poly(amino acid)‐based hydrogels in BTE.^[^ ^10^, ^13^ ^]^ Bioactive glass (BG) has been increasingly applied as an inorganic component in composites due to its bone‐bonding ability and osteogenic properties.^[^ ^14^ ^]^ For instance, the utilization of sub‐micron particles of BG composed of 70% SiO_2_ and 30% CaO has been shown to promote bone cell proliferation and enhance osteogenic differentiation.^[^ ^15^ ^]^ Borosilicate BGs are composed of SiO_2_‐Na_2_O‐CaO‐P_2_O_5_‐B_2_O_3_, which can integrate the excellent properties of silicate and borate BGs. When B_2_O_3_ exists as B‐O triangles, it can reduce the degree of connectivity of the internal spatial network of BGs, which is easier to degrade and more chemically active than Si‐O tetrahedra, and it can accelerate the degradation of borosilicate BGs and effectively improve the shortcomings of traditional silicate BGs that cannot be thoroughly degraded and have a low HA formation rate. The analysis of HA structure formed by boron (B)‐containing and B‐free BGs in simulated body fluids (SBF) demonstrated that B‐containing BGs exhibited accelerated biomineralization rates, leading to the formation of denser and structurally sound HA stacks. Additionally, the presence of boron promoted the nucleation and growth of HA crystals.^[^ ^16^ ^]^ In addition, borosilicate BGs have a large glass‐forming region within them, which can be doped with functional metal elements to confer additional properties to borosilicate BGs. Strontium (Sr) is an alkaline earth metal that can increase bone density and promote bone regeneration by increasing osteoblast activity and inhibiting osteoclast activity.^[^ ^17^ ^]^ Therefore, the utilization of Sr‐doped borosilicate BGs in bone regeneration therapy shows potential. Studies have demonstrated that a 6 mol% concentration of SrO in BGs yields optimal outcomes for repairing bone defects.^[^ ^18^ ^]^ Composite hydrogel vascularization is essential for bone healing. Osteogenic mineralization and neovascularization are closely linked and support each other in bone remodeling.^[^ ^19^ ^]^ However, it has been shown that the proangiogenic effects of Sr‐doped BGs still need to be improved.^[^ ^18c,d^ ^]^ Vascular endothelial growth factor (VEGF) is a major angiogenic factor that promotes vascular growth by facilitating the proliferation and differentiation of vascular endothelial cells, forming blood vessels, and delivering nutrients to the site of regenerating tissue.^[^ ^20^ ^]^ Therefore, the angiogenic factor‐incorporated composite hydrogel serves as a promising biofunctionalized device for bone regeneration.

**Scheme 1:**
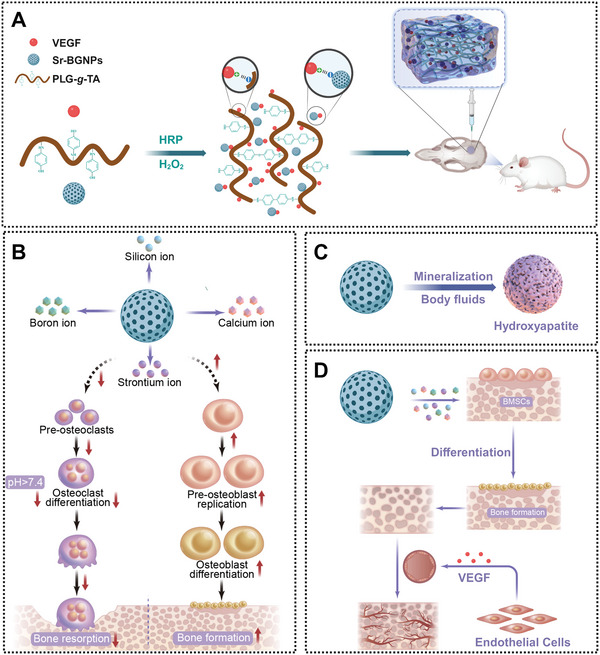
Schematic diagram of the composite hydrogel for synergistic therapy of bone defects. A) Schematic diagram of the preparation of in situ hydrogels and their use in cranial repair. The PLG‐*g*‐TA polymer, VEGF, and Sr‐BGNPs were catalyzed to form PLG‐*g*‐TA/VEGF/Sr‐BGNPs hydrogels in the presence of H_2_O_2_ and HRP, and the composite hydrogels were injected in situ into cranial defects in SD rats to promote their regenerative repair, in which VEGF could be adsorbed to the PLG‐*g*‐TA hydrogel and Sr‐BGNPs via electrostatic forces. B) Sr‐BGNPs can be degraded to release functional ions, of which Sr can play a role in promoting osteoblast differentiation and inhibiting osteoclast differentiation, and the alkaline microenvironment (pH > 7.4) formed by the degradation of Sr‐BGNPs can also effectively inhibit osteoclast differentiation and synergistically play a better role in promoting bone formation. C) Sr‐BGNPs can undergo in situ mimetic mineralization in body fluids by ion exchange with their surroundings without cellular involvement, resulting in the formation of hydroxyapatite. D) The release of VEGF from the composite hydrogels can effectively promote early angiogenesis.

Here, we successfully prepared an inorganic‒organic multifunctional composite hydrogel (PLG‐*g*‐TA/VEGF/Sr‐BGNPs) for synergistic therapy of bone defects through the potentiation of osteogenesis and angiogenesis, as well as the inhibition of osteoclastogenesis (**Scheme** **1**). First, the PLG‐*g*‐TA/VEGF/Sr‐BGNPs hydrogel was synthesized by poly(L‐glutamic acid) (PLG) grafted with tyramine (PLG‐*g*‐TA) polymer, VEGF, and strontium‐doped borosilicate bioactive glass nanoparticles (Sr‐BGNPs) in the presence of hydrogen peroxide (H_2_O_2_) and horseradish peroxidase (HRP), and the physical and chemical properties of the composite hydrogel were tested by fourier transform infrared spectroscopy (FTIR), X‐ray diffraction (XRD), scanning electron microscope (SEM), and inductively coupled plasma‐optical emission spectrometer (ICP‒OES). Then, the composite hydrogels were cocultured with rat bone marrow mesenchymal stem cells (rBMSCs), human umbilical vein endothelial cells (HUVECs), and RAW264.7 cells in vitro to detect their ability to promote osteogenesis and angiogenesis, as well as their ability to inhibit osteoclastogenesis. Finally, the composite hydrogel was implanted into a 5 mm rat cranial bone defect model in vivo to evaluate its bone regeneration ability.

## 2 Results and Discussion

### 2.1 Preparation and Characterization of Sr‐Doped Borosilicate BG Nanoparticles (Sr‐BGNPs)

#### 2.1.1 Structural Analysis of Sr‐BGNPs

**Figure 1:**
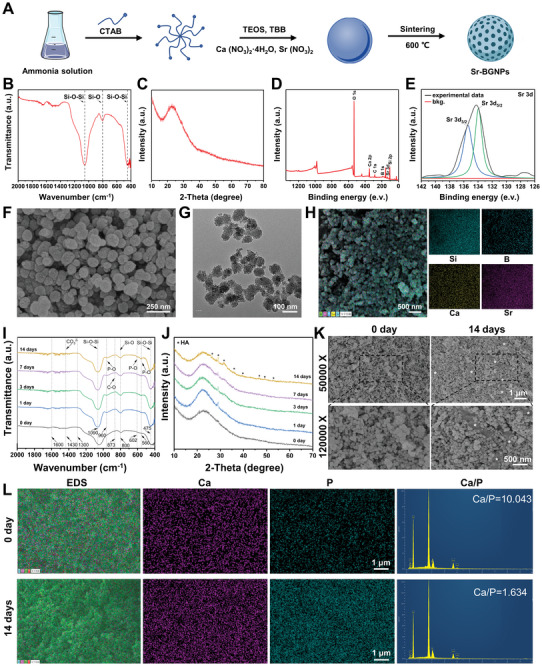
Preparation and characterization of Sr‐BGNPs. A) Diagram of the preparation process of Sr‐BGNPs through the sol–gel method. B) FTIR spectrum of Sr‐BGNPs. C) XRD pattern of Sr‐BGNPs. D) Wide‐survey XPS spectrum of Sr‐BGNPs. E) XPS spectrum of Sr 3d. F) SEM image of the prepared Sr‐BGNPs, which had a nearly regular spherical shape and rough hollow mesoporous structure with a variable particle size in the range of 70 to 100 nm (scale bar = 250 nm). G) TEM image of the prepared Sr‐BGNPs, which were mesoporous dispersed nanoparticles (scale bar = 100 nm). H) EDS mapping showing a uniform distribution of Si, B, Ca, and Sr. I) FTIR spectra of Sr‐BGNPs after immersion in SBF for 1, 3, 7, and 14 days, which showed the presence of apatite. J) XRD patterns of Sr‐BGNPs after immersion in SBF for 1, 3, 7, and 14 days, which showed the presence of apatite. K) SEM images of the surface of Sr‐BGNPs at different magnifications (50 000 X, 120 000 X) after immersion in SBF for 0 and 14 days, which showed the presence of apatite formed by mineralization (*represents newly formed apatite). L) EDS spectra of Sr‐BGNPs nanoparticles after immersion in SBF for 0 and 14 days (scale bar = 1 µm).

The preparation process of the Sr‐BGNPs, which were synthesized in a multicomponent 70SiO_2_‐10B_2_O_3_‐14CaO‐6SrO system using a sol‐gel method, is shown in **Figure** **1**A. FTIR was first applied to examine the structure of the prepared Sr‐BGNPs (Figure 1B). The FTIR spectrum suggested several absorbance bands, in which the peak at 1057 cm^−1^ was attributed to the asymmetric stretching vibration of Si–O–Si, the peak at 803 cm^−1^ was associated with the symmetric stretching vibration of Si–O, and the peak at 447 cm^−1^ was attributed to the symmetric bending vibration of Si–O–Si. XRD analysis was used to detect potential crystalline phases of the prepared Sr‐BGNPs, and the results showed a broad peak at 23° (2θ), indicating the amorphous structure of the Sr‐BGNPs (Figure 1C). XPS was used to examine the Si, B, Ca, and Sr incorporated in the network of Sr‐BGNPs (Figure 1D). The results further confirmed that the peaks of the Sr 3d_5/2_ and Sr 3d_3/2_ electrons were located at 133.98 eV and 135.38 eV, respectively, for the Sr‐BGNPs (Figure 1E). The microscopic morphology of the Sr‐BGNPs was observed using SEM and transmission electron microscope (TEM). As shown in Figure 1F,G, the Sr‐BGNPs showed good monodispersity, nearly regular spherical shapes, and rough hollow mesoporous structures on the surface, with particle sizes ranging from 70 to 100 nm. EDS mapping revealed a uniform distribution of silicon (Si), boron (B), calcium (Ca), and Sr (Figure 1H). A zeta potential test confirmed that the Sr‐BGNPs exhibited a negative charge of −13.04 ± 0.33 mV (Figure S1, Supporting Information). The zeta potential of BGNPs affects their biological activity. Amirhossein et al. showed that BGNPs with a negative charge could enhance osteoblast adhesion, proliferation, and osteogenic differentiation.^[^ ^18^, ^21^ ^]^

#### 2.1.2 The Apatite‐Forming Ability of Sr‐BGNPs

Mineralization of Sr‐BGNPs in SBF was detected by FTIR, XRD, and SEM in vitro.^[^ ^22^ ^]^ After 14 days of immersion in SBF, the characteristic absorption peaks and crystalline diffraction peaks of apatite appeared in FTIR and XRD patterns, respectively. The newly observed double shoulder peaks at 560 and 602 cm^−1^ were attributed to the bending vibration of [P‐O], the peak at 960 cm^−1^ was attributed to the stretching vibration of [P‐O], and the peaks at 873 and 1430 cm^−1^ were attributed to [CO_3_^2−^] (Figure 1I). Moreover, the characteristic crystalline diffraction peaks that appeared on the XRD pattern were compared with those of the HA standard card (JCPDS 09–0432), and the results showed that the new peaks that appeared at 2θ values of 53°, 49°, 46°, 39°, 32°, and 26° could correspond to the (004), (213), (222), (310), (211), and (002) crystalline surfaces on the HA structure, respectively (Figure 1J).^[^ ^23^ ^]^ In addition, the intensity of each characteristic absorption peak on FTIR and each crystalline diffraction peak on XRD gradually increased with increasing soaking time, indicating that the Sr‐BGNPs mineralized to form more apatite. SEM images showed that apatite was mainly located between the voids formed by the stacking of Sr‐BGNPs, which was related to the high concentration of Ca^2+^ released by the degradation of Sr‐BGNP interparticles, as well as the negative curvature of the surface of the Sr‐BGNPs (Figure 1K). It was shown that when the surface curvature of the material was negative, the OH—O spacing between two neighboring hydroxyl groups decreased, which made it easy to form hydrogen bonds, resulting in a stronger bond between the hydroxyl groups and the surface of the material and an increase in the number of silica‐hydroxyl groups and nucleation sites for the formation of apatite by mineralization.^[^ ^24^ ^]^ In addition, a gradual increase in phosphorus (P) could be seen in the EDS mapping at Days 0 and 14, and the Ca/P ratio on Day 14 was approximately 1.634, which was close to the stoichiometric Ca/P ratio of 1.67 for HA in human bone, indicating the formation of HA (Figure 1L). To date, Sr‐BGNPs were well prepared in this study, and they could induce apatite formation, showing potential as bone repair candidates.

### 2.2 Preparation and Characterization of the PLG‐*g*‐TA/VEGF/Sr‐BGNPs Hydrogel

#### 2.2.1 Formation of the PLG‐g‐TA Hydrogel and Measurement of Gelation Time

**Figure 2:**
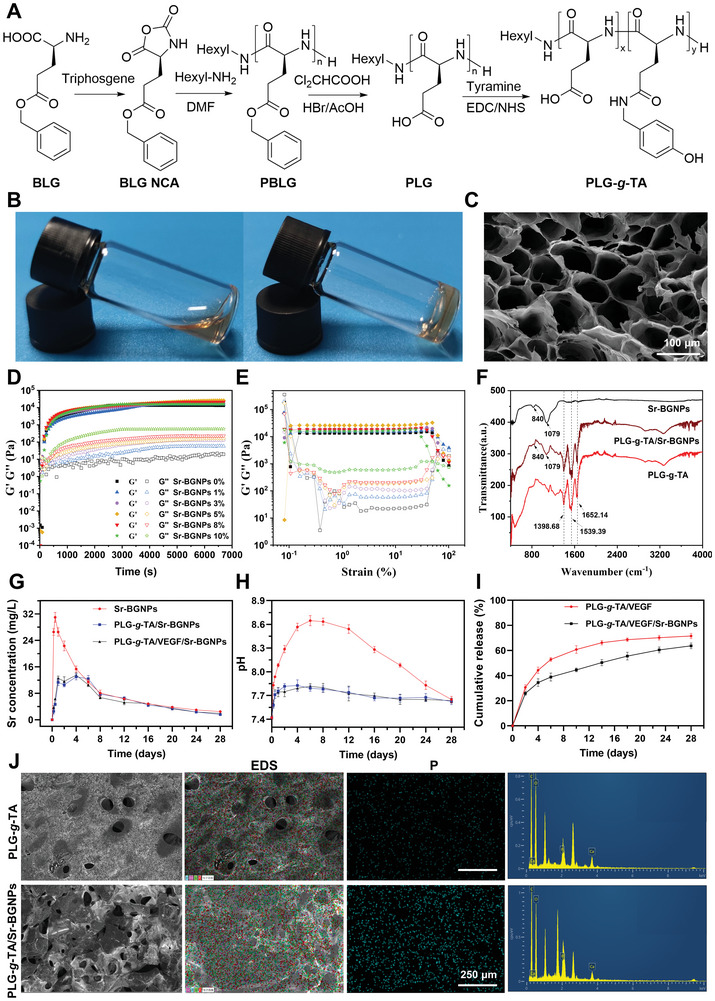
Preparation and characteristics of the PLG‐*g*‐TA/VEGF/Sr‐BGNPs hydrogel. A) The synthetic route of the PLG‐g‐TA copolymer. B) The vial inversion method was used to determine the gel‐forming ability. The mixed solution was fluid before gel formation and lost its fluidity after gel formation. C) SEM images of the PLG‐*g*‐TA hydrogels (scale bar = 100 µm). The hydrogels were formed with a final concentration of 5% (w/v) of the PLG‐*g*‐TA copolymer, 8 units/mL of HRP, and H_2_O_2_ (nH_2_O_2_/nTA = 0.4). D,E) Rheological experiments of the hydrogels. The G’ and G’’ moduli of the PLG‐*g*‐TA and PLG‐*g*‐TA/Sr‐BGNPs hydrogels measured in a time‐dependent model (D) and shear strain test (E). F) FTIR spectra of monodisperse Sr‐BGNPs, the PLG‐*g*‐TA hydrogel, and the PLG‐*g*‐TA/Sr‐BGNPs hydrogel. The hydrogels formed by a final concentration of 5% (w/v) of PLG‐*g*‐TA copolymer, 8 units/mL of HRP, H_2_O_2_ (nH_2_O_2_/nTA = 0.4), and 5% (w/v) of Sr‐BGNPs. G,H) Concentration of Sr (G) ions and the pH (H) change in the medium after 0.25, 0.5, 1, 2, 4, 6, 8, 12, 16, 20, 24, and 28 days of immersion in SBF for monodisperse Sr‐BGNPs and composite hydrogels loaded with Sr‐BGNPs (straight lines represent changes in concentration trends, *n* = 3 per group). I) Cumulative release of VEGF from the PLG‐*g*‐TA/VEGF and PLG‐*g*‐TA/VEGF/Sr‐BGNPs hydrogels using the ELISA method (*n* = 3 per group). J) SEM images and element distribution of PLG‐*g*‐TA/VEGF and PLG‐*g*‐TA/Sr‐BGNPs hydrogels after 7 days of mineralization in SBF solution (scale bar = 250 µm).

The PLG‐*g*‐TA copolymer was synthesized from PLG conjugated with tyramine (TA). As shown in **Figure** **2**A, and l‐glutamic acid γ‐benzyl ester (BLG) was used as the raw material, and PLG was obtained by synthesizing the intermediates BLG‐NCA and PBLG and was finally deprotected. The ^1^H NMR spectra of BLG‐NCA, PBLG, PLG, and PLG‐*g*‐TA are shown in Figure S2–S5 (Supporting Information), with the peaks assigned.^[^ ^25^ ^]^ As illustrated in Figure S6 (Supporting Information), the injectable PLG‐*g*‐TA hydrogel was synthesized via an enzymatically cross‐linked reaction with HRP and H_2_O_2_.^[^ ^26^ ^]^ The vial inversion method was used to determine the gel‐forming ability and gel‐forming time of the PLG‐*g*‐TA hydrogel (Figure 2B).^[^ ^27^ ^]^ The results showed that the gelation time ranged from a few seconds to several minutes, as determined by the concentrations of H_2_O_2_, HRP, and the PLG‐*g*‐TA copolymer (Figure S7, Supporting Information).

#### 2.2.2 Morphology and Degradation of the PLG‐g‐TA Hydrogel

The microscopic morphology of the PLG‐*g*‐TA hydrogel was observed via SEM. As shown in Figure 2C, the PLG‐*g*‐TA hydrogels exhibited interconnected honeycomb‐like continuous macropores with pore diameters ranging from 80–100 µm, accompanied by some uniformly dispersed tiny pores with diameters ranging from 10–20 µm. Furthermore, the average porosity was 60.38 ± 1.18%. Thus, the hydrogel constructed in this study had a suitable pore size for cell growth and the ability to load drugs or bioactive molecules. In vitro enzymatic degradation of PLG‐*g*‐TA was performed in PBS containing 2 mg mL^−1^ elastase. The change in their weight was mainly affected by the combined effect of degradation and swelling. The results showed that the mass of the hydrogel increased after 2 days of immersion, which was caused by swelling of the hydrogel (Figure S10, Supporting Information). However, in the presence of elastase, the hydrogel showed a mass loss beginning on Day 6, with 75.74 ± 3.51% of the mass remaining on Day 22, 49.74 ± 1.76% on Day 26, and almost complete degradation on Day 29. The in vivo degradation of the hydrogel subcutaneously in rats was related to the enzymatic degradation of amide bonds present in the polyglutamic acid structure by naturally occurring proteases in rats. As shown in Figure S11 (Supporting Information), the volume of the hydrogel gradually decreased with increasing time after subcutaneous implantation, indicating that the hydrogel could gradually degrade in rats, and the hydrogel almost completely disappeared 8 weeks after injection. Moreover, hematoxylin and eosin (H&E) staining results showed that with the gradual degradation of the hydrogel, the number of immune cells gradually decreased. The number of immune cells in the tissue sections was not significantly different from that in the normal skin tissue sections after 8 weeks of implantation, demonstrating that the hydrogel has degradability and good biological safety in vivo (Figure S12, Supporting Information).

#### 2.2.3 Rheological Properties and FTIR Spectrum of the PLG‐g‐TA/Sr‐BGNPs Hydrogel

To further evaluate the effect of the incorporation of Sr‐BGNPs on the storage (G′) and loss (G″) moduli of the composite hydrogel, the gel‐forming kinetics, shear strain, and frequency scans of the composite hydrogels with Sr‐BGNP contents of 1, 3, 5, 8, and 10% (w/v) were tested. As shown in Figure 2D, the maximum plateau G′ of the PLG‐*g*‐TA hydrogel was 13 566 Pa. The G′ and G” of the composite hydrogel increased with increasing amounts of incorporated Sr‐BGNPs, and when the amount of incorporated Sr‐BGNPs was 5% (w/v), the maximum platform G′ was 26 127 Pa, and the maximum platform G″ was 170.56 Pa. However, when the amount of incorporated Sr‐BGNPs was 8% or 10% (w/v), the maximum platform G′ decreased, with a10% decrease most significantly. The appropriate content (5%) of incorporated Sr‐BGNPs could be better dispersed in the composite hydrogel, but when the amount of incorporated Sr‐BGNPs was too high, the dispersion was poor, which directly led to a decrease in the rigidity of the composite hydrogel. The shear strain results are shown in Figure 2E. When the amount of incorporated Sr‐BGNPs was greater than 5%, the maximum platform G′ decreased with increasing of Sr‐BGNPs concentration, the gel integrity decreased in advance, and the mechanical strength decreased rapidly, which was consistent with the results of the gel formation kinetics. The frequency scanning results are shown in Figure S13 (Supporting Information). When the amount of incorporated Sr‐BGNPs exceeded 5%, the composite hydrogel was destroyed at more minor test frequencies, and the platform modulus decreased in advance. In summary, 5% (w/v) Sr‐BGNPs were selected for the construction of the composite hydrogel in a subsequent study.

The FTIR spectra of Sr‐BGNPs, PLG‐*g*‐TA, and PLG‐*g*‐TA/Sr‐BGNPs showed that Sr‐BGNPs were successfully added to the hydrogel, with peaks at 840 and 1079 cm^−1^ representing the absorption peaks of Si‐O and Si‐O‐Si, respectively. Moreover, the same peaks at 1398.68, 1539.39, and 1652.14 cm^−1^ were still maintained, which showed that the loading of Sr‐BGNPs did not have any obvious effect on the main functional groups of the PLG‐*g*‐TA hydrogels (Figure 2F).

#### 2.2.4 The Release of Ions from the PLG‐g‐TA/VEGF/Sr‐BGNPs Hydrogel In Vitro

The ions released from the Sr‐BGNPs, PLG‐*g*‐TA/Sr‐BGNPs, and PLG‐*g*‐TA/VEGF/Sr‐BGNPs hydrogels were investigated by ICP‒OES, and the pH change during mineralization was also measured. As shown in Figure 2G and Figure S14 (Supporting Information), when the Sr‐BGNPs were immersed in SBF, they gradually released Si, Ca, B, and Sr ions. Supersaturated Ca^2+^ and PO_4_^3−^ were deposited on the surface of the Sr‐BGNPs to form HA, which in turn inhibited further ion release from the Sr‐BGNPs. Therefore, both B and Sr showed a rapid increase in the initial 48 h, followed by a gradual decrease. The trends of the ion concentrations of PLG‐*g*‐TA/Sr‐BGNPs and PLG‐*g*‐TA/VEGF/Sr‐BGNPs were similar over time. Comparing the release curves with those of Sr‐BGNPs, the peak concentration of each ion in the composite hydrogel group decreased, and the time for the ion concentration to reach the peak was prolonged. For example, compared with that of Sr‐BGNPs, the time to reach the peak concentration of Sr was delayed on Day 4, and their maximum concentrations decreased by 57.955 ± 3.925% and 56.332 ± 2.413%, respectively (Figure 2G). Overall, the hydrogels slowed the degradation process of Sr‐BGNPs, effectively avoiding the rapid increase in the ion concentration caused by the release of a large number of ions at the early stage, and were able to maintain a stable ion concentration in the environment for a long period.

#### 2.2.5 Changes in the pH of the PLG‐g‐TA/VEGF/Sr‐BGNPs Hydrogel In Vitro

The pH changes in the environment are shown in Figure 2H. The pH of the monodisperse Sr‐BGNPs peaked on approximately Day 6, at ≈8.646 ± 0.052, and then the pH slowly decreased with time, reaching 7.650 ± 0.032 on Day 28. The pH changes of the PLG‐*g*‐TA/Sr‐BGNPs and PLG‐*g*‐TA/VEGF/Sr‐BGNPs hydrogels had similar trends, both showing an early rapid increase and peaking on Days 2–4, at 7.826 ± 0.057 and 7.81 ± 0.029, respectively, followed by a slow decrease and remaining at 7.61–7.65. The environmental acidity and alkalinity influence the activity of osteoblasts and osteoclasts. Alkaline phosphatase (ALP) activity and collagen deposition processes, closely related to bone mineralization, are inhibited under acidic conditions, while osteoclast activity is enhanced, ultimately leading to bone loss.^[^ ^28^ ^]^ Harada et al. showed that ALP activity was elevated by 1.667‐fold when the pH increased from 7.4 to 8.5. In contrast, its activity decreased by up to 10‐fold when the pH decreased from 7.4 to 6.9, and a suitably alkaline environment increased ALP activity, which facilitated the formation of calcium nodules and promoted osteogenesis.^[^ ^29^ ^]^ Cui et al. cocultured MSCs with media at different pH values (6.750–7.950) for 1–7 days and found that MSCs could maintain growth and proliferation activity only at pH 7.350‐7.800, whereas outside of this range, cell proliferation was inhibited, and the survival rate decreased.^[^ ^30^ ^]^ In addition, overactivated osteoclast activity could be effectively corrected in an alkaline environment. Liu et al. showed that when osteoclasts were cultured at pH ≥ 7.8, the expression of osteoclast‐related genes, such as histone K, Trap, MMP9, and NFATc1, was suppressed, and their osteoblastic differentiation and bone resorption ability were also significantly inhibited.^[^ ^31^ ^]^ Therefore, a suitable alkaline environment can effectively promote osteoblast proliferation, increase ALP activity, and enhance osteogenic differentiation while inhibiting osteoclast activity, providing a new idea for treating osteoclast‐related disorders, which could be achieved by creating a relatively alkaline microenvironment through the degradation of the implant itself and inhibiting the activity of abnormal osteoclasts.

#### 2.2.6 The Release of VEGF from the PLG‐g‐TA/VEGF/Sr‐BGNPs Hydrogel In Vitro

The VEGF release of the PLG‐*g*‐TA/VEGF and PLG‐*g*‐TA/VEGF/Sr‐BGNPs hydrogels was examined by enzyme‐linked immunosorbent assay (ELISA). As shown in Figure 2I, the PLG‐*g*‐TA/VEGF and PLG‐*g*‐TA/VEGF/Sr‐BGNPs hydrogels showed biphasic release characteristics, manifesting as initial explosive release and subsequent slow release. Therefore, the release of VEGF from the PLG‐*g*‐TA/VEGF and PLG‐*g*‐TA/VEGF/Sr‐BGNPs hydrogels was stable for a long period of time. Moreover, the slow‐release trend of the PLG‐*g*‐TA/VEGF/Sr‐BGNPs group was similar to that of the PLG‐*g*‐TA/VEGF group, but its release amount at the same time was less than that of the PLG‐*g*‐TA/VEGF group, demonstrating that the PLG‐*g*‐TA/VEGF/Sr‐BGNPs hydrogel had a better slow‐release ability. The isoelectric point of hVEGF^165^ was reported to be 8.5,^[^ ^32^ ^]^ and the pH fluctuation of the PLG‐*g*‐TA/VEGF/Sr‐BGNPs group in SBF was in the range of 7.61–7.82. Therefore, the VEGF released into PBS and compounded in the hydrogel were both positively charged and could interact with the negatively charged Sr‐BGNPs via electrostatic interactions to slow their release. Moreover, the hollow mesoporous structure inside the Sr‐BGNPs could also load a certain amount of VEGF, thus realizing the dual‐release retardation effect.^[^ ^33^ ^]^

#### 2.2.7 The Apatite‐Forming Ability of the PLG‐g‐TA/Sr‐BGNPs Hydrogel In Vitro

To evaluate the in vitro mineralization ability of the composite hydrogels, hydrogels were lyophilized for SEM after 7 days of immersion. As shown in Figure 2J, the surface of the PLG‐g‐TA/Sr‐BGNPs hydrogel was rough with visible HA deposits, indicating that the composite hydrogel loaded with Sr‐BGNPs was able to mineralize and form HA in vitro. The EDS results showed that the surface deposits of the PLG‐*g*‐TA/Sr‐BGNPs hydrogel were enriched with apparent P, which also reflected the occurrence of mineralization.

### 2.3 Cell Proliferation and Cell Migration Assays In Vitro

**Figure 3:**
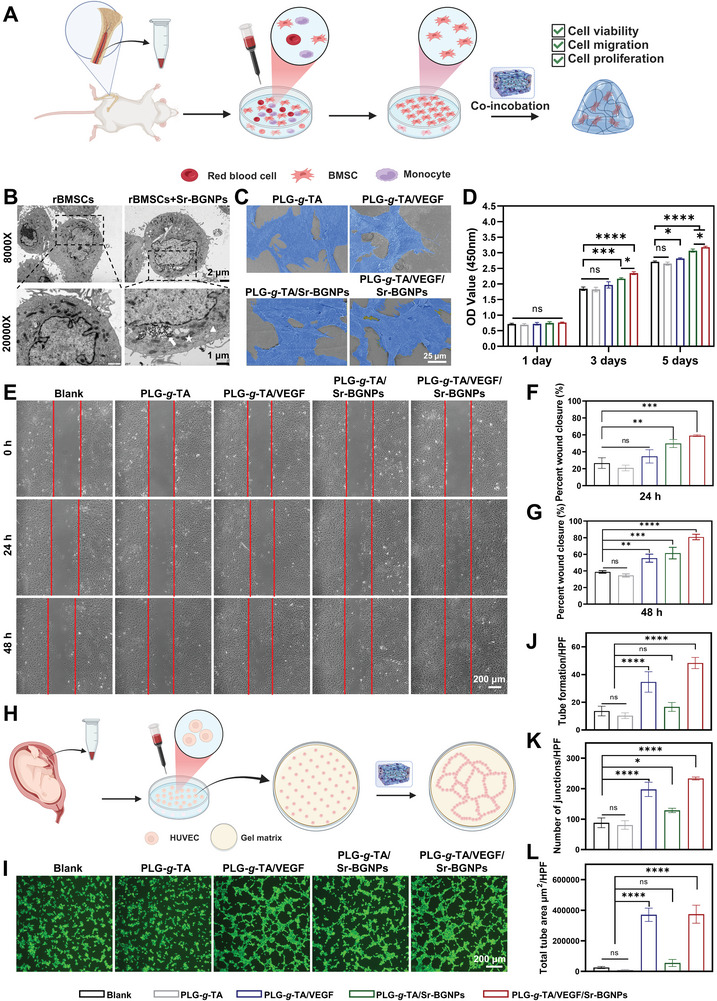
Cytocompatibility, proliferation, migration, and angiogenesis of the PLG‐*g*‐TA/VEGF/Sr‐BGNPs hydrogel in vitro. A) Schematic diagram of rBMSC extraction and evaluation in vitro. B) TEM images of rBMSCs and rBMSCs phagocytosed with Sr‐BGNPs, which showed no obvious toxicity in the internal organelle structure of rBMSCs phagocytosed with Sr‐BGNPs compared with the control group, and Sr‐BGNPs had good biosafety (white arrows refer to Sr‐BGNPs phagocytosed by the cells, pentagrams refer to cellular microstructure mitochondria, triangles refer to endoplasmic reticulum). C) SEM images of cell attachment after culturing for 48 h. D) Proliferation of cells cultured with five groups for 1, 3 and 5 days. E) Representative images of scratch healing for cell migration for 24 and 48 h (the red line represents the boundaries of each group of the scratch at the initial 0 h, scale bar = 200 µm). F) Quantitative analysis of cell migration after 24 h. G) Quantitative analysis of cell migration after 48 h. H) Schematic diagram of tube formation assay in vitro. I) Representative images of the tube formation assay of HUVECs stimulated for 2 h (scale bar = 200 µm). J) Quantitative analysis of tube formation number per high power field (HPF). K) Quantitative analysis of junction's number per HPF. L) Quantitative analysis of total tube area per HPF. The data in Figure 3D, F, G, J–L were analyzed using one‐way ANOVA, and were presented as the means ± SDs. Asterisks indicate *p* values, *p < 0.05, **p < 0.01, ***p < 0.001, ****p < 0.0001, and ns represents no significant difference. *n* = 3 per group.

RBMSCs isolated from the tibial bone marrow of Sprague‒Dawley (SD) rats were used to test the in vitro biocompatibility of the PLG‐*g*‐TA/VEGF/Sr‐BGNPs hydrogel (**Figure** **3**A). As shown in Figure S15A,B (Supporting Information), the extracted cells had a typical spindle‐shaped morphology, and the flow cytometry results revealed the expression of the MSC markers CD29 (99.4%) and CD90 (99.8%), with the absence of the hematopoietic cell markers CD34 (6.0%) and CD45 (6.4%). In addition, the cells showed potential for multidirectional differentiation into osteogenic, chondrogenic, and adipogenic lineages (Figure S15C, Supporting Information). The cytocompatibility of the PLG‐*g*‐TA copolymer was assessed by a CCK‐8 assay (Figure S16, Supporting Information), while the cytocompatibility of the Sr‐BGNPs was assessed by TEM (Figure 3B) and hemolysis tests (Figure S17, Supporting Information). RBMSCs were further observed by SEM after 48 h of coculture on the surface of the PLG‐*g*‐TA, PLG‐*g*‐TA/VEGF, PLG‐*g*‐TA/Sr‐BGNPs, and PLG‐*g*‐TA/VEGF/Sr‐BGNPs hydrogels. As shown in Figure 3C, the cells were able to adhere and grow efficiently on the surface of the four hydrogels, spreading out and extending a large number of cellular pseudopods, which was closely related to the fact that the hydrogels mimicked the natural ECM structure with interpenetrating porous structures and high water content, which normally promotes the adhesion of cells.^[^ ^34^ ^]^ The cytocompatibility of the PLG‐*g*‐TA/VEGF/Sr‐BGNPs hydrogel was further evaluated via live/dead staining. As shown in Figure S18 (Supporting Information), a large proportion of cells survived in the five groups, with only a few dead cells stained red. In addition, the CCK‐8 assay was used to detect the proliferation of the rBMSCs. As shown in Figure 3D, after 1 day of coculture, the difference in cell proliferation of the remaining four hydrogel groups was not statistically significant (*p* > 0.05) compared to that of the blank group; after 3 days of coculture, compared with that of the blank group, the cell proliferation of the PLG‐g‐TA group was weakened and that of the PLG‐*g*‐TA/Sr‐BGNPs and PLG‐*g*‐TA/VEGF/Sr‐BGNPs groups had significant cell proliferation, and the difference was statistically significant (*p* < 0.001), which was considered to be caused by the combination of slow‐release of Sr^2+^ and prolonged action of VEGF. Although the proliferation ability of cells in the PLG‐*g*‐TA/VEGF group was increased compared with that in the blank group, the difference between groups was not statistically significant (*p* > 0.05). After 5 days of coculture, cell proliferation was still obvious in the PLG‐*g*‐TA/Sr‐BGNPs and PLG‐*g*‐TA/VEGF/Sr‐BGNPs groups, and the PLG‐*g*‐TA/VEGF/Sr‐BGNPs group had the strongest proliferative ability.

It is well known that BMSCs play a crucial role in the bone healing process. The recruitment of MSCs with osteogenic differentiation potential into bone defects is an essential indicator for testing whether the material has excellent osteogenic properties.^[^ ^35^ ^]^ Therefore, cell scratch experiments were performed to evaluate the ability of the PLG‐*g*‐TA/VEGF/Sr‐BGNPs hydrogel to recruit MSCs. As shown in Figure 3E, the remaining scratch area of each group gradually decreased over time. Compared with that of the other groups, the cell migration area of the PLG‐g‐TA/VEGF/Sr‐BGNPs group was the largest, which indicated that PLG‐*g*‐TA/VEGF/Sr‐BGNPs promoted the migration of rBMSCs at an early stage and laid a foundation for subsequent osteogenic differentiation. In addition, at 24 h, although the cell migration area of the PLG‐*g*‐TA/VEGF group was greater than that of the blank group, the difference was not statistically significant (*p* > 0.05) (Figure 3F). However, the difference between the two groups was statistically significant at 48 h (*p* < 0.01), demonstrating that VEGF also promoted the migration of the rBMSCs over time (Figure 3G). Midy et al. reported that VEGF could play a role in the differentiation of osteoblasts by enhancing the migration ability of rBMSCs and the ALP activity of osteoblasts, but their study concluded that VEGF could not effectively promote the proliferation of rBMSCs.^[^ ^36^ ^]^ Compared with other studies showing that VEGF could promote the proliferation of rBMSCs, it was speculated that 20 h of coculture stimulation, according to the study of Midy et al., did not significantly affect the proliferation of rBMSCs. In our study, although differences between the PLG‐*g*‐TA/VEGF, PLG‐*g*‐TA, and blank groups in promoting cell proliferation were not statistically significant on Days 1 and 3 (*p* > 0.05), the promotional effect of the PLG‐*g*‐TA/VEGF group was evident on Day 5 (*p* < 0.05). Therefore, the effect of VEGF on the proliferation of rBMSCs should not be ignored.

### 2.4 Angiogenesis of the PLG‐*g*‐TA/VEGF/Sr‐BGNPs Hydrogel In Vitro

Osteogenesis and angiogenesis are two inseparable processes in bone healing, and bone graft materials constructed by BTE should have good angiogenic ability in addition to good osteogenic properties. Early angiogenesis is a crucial process for successful bone regeneration.^[^ ^37^ ^]^ VEGF is the most potent bioactive factor known to promote neovascularization and can specifically act on endothelial cells in vitro to promote blood vessel formation and vascularization of newborn tissues in vivo, which can directly or indirectly affect bone regeneration.^[^ ^38^ ^]^ As the most direct evidence of angiogenesis, a tube formation assay was conducted to evaluate the angiogenic ability of HUVECs by coculturing the cells in five groups (Figure 3H). As shown in Figure 3I, obvious tube formation appeared in the PLG‐*g*‐TA/VEGF, PLG‐*g*‐TA/Sr‐BGNPs, and PLG‐*g*‐TA/VEGF/Sr‐BGNPs groups at 2 h. In addition, the number of tubes formed (Figure 3J), the number of junctions (Figure 3K), the total area (Figure 3L), and the total length (Figure S19, Supporting Information) in the PLG‐*g*‐TA/VEGF and PLG‐*g*‐TA/VEGF/Sr‐BGNPs groups were significantly greater than those in the blank, PLG‐*g*‐TA, and PLG‐*g*‐TA/Sr‐BGNPs groups (*p* < 0.05). Therefore, the PLG‐*g*‐TA/VEGF/Sr‐BGNPs hydrogel had excellent angiogenic properties.

### 2.5 Osteogenic Differentiation In Vitro

Both Sr^2+^ and BG can promote the osteogenic differentiation of rBMSCs.^[^ ^18^, ^39^ ^]^ Pierre et al. proposed that Sr^2+^ activated calcium‐sensitive receptor (CaSR) to stimulate bone formation by increasing the production of prostaglandin E_2_ (PGE_2_) by osteoblasts.^[^ ^40^ ^]^ In addition to stimulating the CaSR signaling pathway, Yang et al. demonstrated that Sr^2+^ could stimulate β‐catenin expression in vitro and in vivo by upregulating ECM gene expression and activating the Wnt/β‐catenin pathway to enhance MSC osteogenic differentiation and bone formation in vivo.^[^ ^41^ ^]^ Huang et al. showed that Sr‐doped BG could synergistically activate the Wnt/β‐catenin pathway in MSCs through the degradation of released Sr and Si to promote bone formation.^[^ ^42^ ^]^

**Figure 4:**
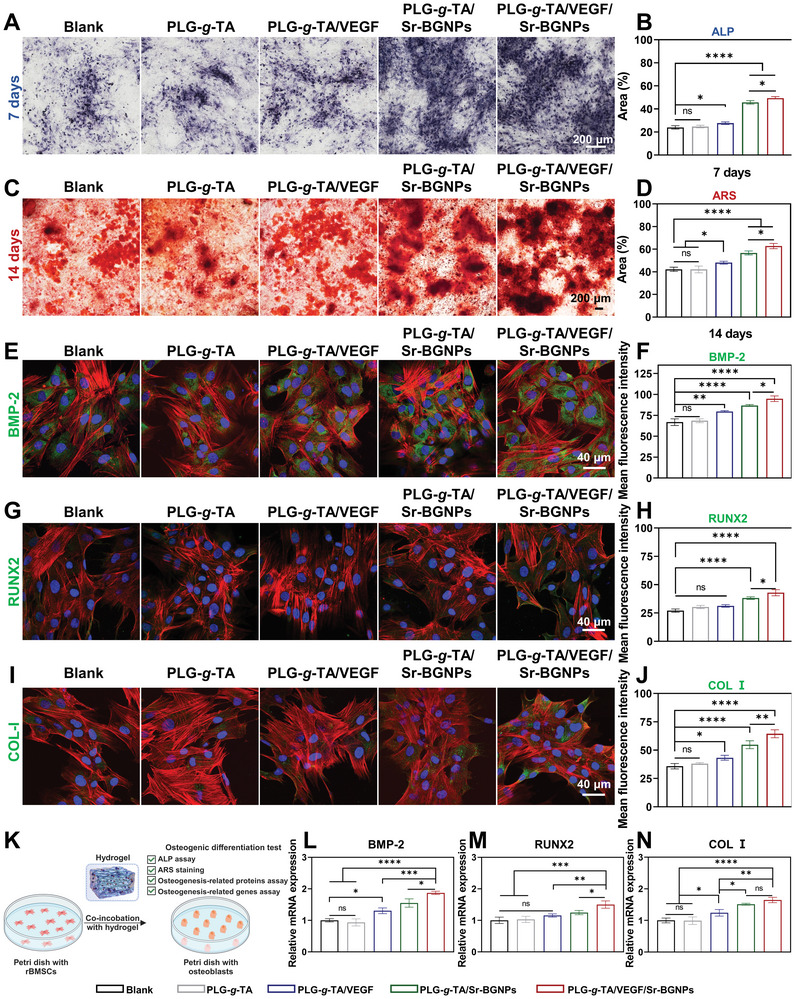
Osteogenic differentiation of the PLG‐g‐TA/VEGF/Sr‐BGNPs hydrogel in vitro. A) ALP staining of rBMSCs cultured for 7 days (scale bar = 200 µm). B) Quantitative analysis of the ALP staining area. C) ARS staining of rBMSCs cultured for 14 days (scale bar = 200 µm). D) Quantitative analysis of the ARS area. E) Representative immunofluorescence images of BMP‐2 (green) (scale bar = 40 µm) on Day 14. F) Quantitative analysis of the mean fluorescence intensity of BMP‐2. G) Representative immunofluorescence images of RUNX2 (green) (scale bar = 40 µm) on Day 14. H) Quantitative analysis of the mean fluorescence intensity of RUNX2. I) Representative immunofluorescence images of COL‐I (green) (scale bar = 40 µm) on Day 14. J) Quantitative analysis of the mean fluorescence intensity of COL‐I. K) Schematic diagram of osteogenic activity from the PLG‐g‐TA/VEGF/Sr‐BGNPs in vitro. L–N) Quantitative analysis of the expression of the osteogenic genes BMP‐2 (L), RUNX2 (M), and ALP (N) in rBMSCs on Day 14. The data in Figure 4B, D, F, H, J, L‐N were analyzed using one‐way ANOVA, and were presented as the means ± SDs. Asterisks indicate *p* values, *p < 0.05, **p < 0.01, ***p < 0.001, ****p < 0.0001, and ns represents no significant difference. *n* = 3 per group.

ALP can be used to detect osteogenic differentiation at an early stage, while alizarin red staining (ARS) can be used to detect cell mineralization at a late differentiation stage.^[^ ^43^ ^]^ First, ALP staining was performed, and the percentage of the positive area was quantitatively analyzed using ImageJ software after the rBMSCs were cocultured with the five groups in osteogenic induction medium for 7 days. As shown in **Figure** **4**A, compared with those in the other four groups, the ALP expression in the rBMSCs of the PLG‐g‐TA/VEGF/Sr‐BGNPs group was significantly greater, and the PLG‐*g*‐TA/VEGF/Sr‐BGNPs group had the largest percentage of positive area (Figure 4B). ARS was performed to evaluate calcium deposition after the rBMSCs were cocultured with the five groups in osteogenic induction medium for 14 days. As shown in Figure 4C, compared with the other four groups, the PLG‐g‐TA/VEGF/Sr‐BGNPs group exhibited significantly greater calcium deposition in the rBMSCs and had the largest percentage of positive area (Figure 4D).

Then, the relative expression of osteogenesis‐related proteins (BMP‐2, RUNX2, and COL I) was detected using immunofluorescence staining after the rBMSCs were cocultured with the five groups in osteogenic induction medium for 14 days. As shown in Figure 4E–J, the PLG‐*g*‐TA/VEGF/Sr‐BGNPs significantly upregulated the BMP‐2 (Figure 4E,F), RUNX2 (Figure 4G,H), and COL I (Figure 4I,J) protein levels compared to those in the other four groups. Finally, the relative expression of osteogenesis‐related genes (BMP‐2, RUNX2, and ALP) was detected using RT‒PCR after the rBMSCs were co‐cultured with the five groups in osteogenic induction medium for 14 days. As shown in Figure 4L–N, the PLG‐*g*‐TA/VEGF/Sr‐BGNPs significantly upregulated the BMP‐2 (Figure 4L), RUNX2 (Figure 4M), and ALP (Figure 4N) gene levels compared to those in the other four groups.

Taken together, the above results demonstrated that the PLG‐*g*‐TA/VEGF/Sr‐BGNPs hydrogel enhanced the osteogenic differentiation of rBMSCs in vitro.

### 2.6 Inhibition of RNAKL‐Induced Osteoblastic Differentiation and Bone Resorption In Vitro

RAW264.7 cells can form mature osteoclasts (nuclei number ≥ 3) after being induced with 50 ng mL^−1^ RANKL.^[^ ^44^ ^]^ The erosive and destructive effects of osteoclasts affect the quality of new bone generation in the area of bone defects. Sr^2+^ has been shown to inhibit osteoclast differentiation and up‐regulate the expression of osteoprotegerin (OPG) to inhibit osteoclast differentiation and maturation, which is based on the principle that OPG is a soluble decoy receptor for RANKL, that inhibits the binding of RANKL to RANK and inhibits the downstream NF‐KB1/ERK1/2 and MAPK/ERK1/2‐mediated transcription of NFATc1, thus inhibiting osteoclast formation.^[^ ^40^ ^]^ Sr‐doped BG inhibited the p38 and NFκB signaling pathways in Raw264.7 cells to inhibit osteogenic differentiation.^[^ ^42^ ^]^ Moreover, environmental acidity and alkalinity influence the activity of osteoclasts. Liu et al. showed that when osteoclasts were cultured at pH ≥ 7.8, the expression of osteoclast‐related genes, such as histone K, Trap, MMP9, and NFATc1, was all suppressed, and their osteoblastic differentiation and bone resorption ability were also significantly inhibited.^[^ ^31^ ^]^ Therefore, a suitable alkaline environment can effectively inhibit osteoclast activity, providing a new idea for treating osteoclast‐related disorders, which could be achieved by creating a relatively alkaline microenvironment through the degradation of the implant itself and inhibiting the activity of abnormal osteoclasts. The PLG‐*g*‐TA/VEGF/Sr‐BGNPs can slow the release of Sr^2+^ and construct an alkaline microenvironment by binding released BO_3_^3‐^ and SiO_4_^4−^ to H^+^ in the environment. Therefore, PLG‐*g*‐TA/VEGF/Sr‐BGNPs can inhibit osteoclast differentiation and maturation.

**Figure 5:**
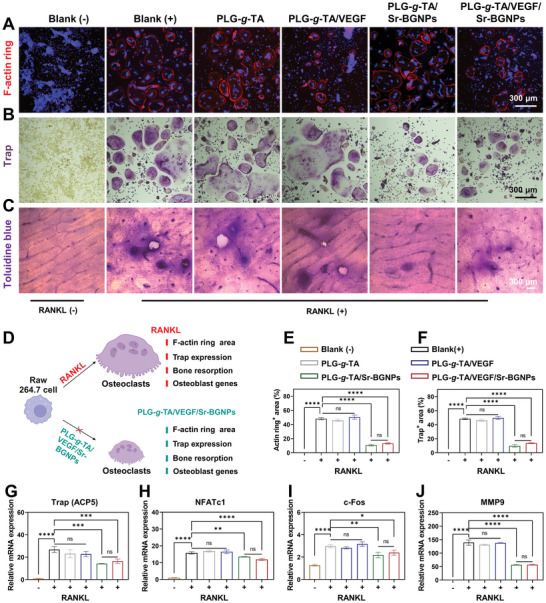
The inhibition of osteoclast differentiation and bone resorption in vitro. A) Representative images of F‐actin ring staining of osteoclasts (scale bar = 300 µm). B) Representative images of TRAP staining of osteoclasts (scale bar = 300 µm). C) Representative images of toluidine blue staining of bovine bone slices (scale bar = 300 µm). D) Schematic of the inhibition of osteoclast differentiation and bone resorption in vitro. E) Quantitative analysis of the area of the actin ring of osteoclasts. F) Quantitative analysis of the area of TRAP staining of osteoclasts. G–J) Quantitative analysis of the expression of the osteoclast differentiation genes Acp5 (G), NFATc1 (H), c‐Fos (I), and MMP‐9 (J) in the different groups cultured for 7 days. The data in Figure 5E‐J were analyzed using one‐way ANOVA, and were presented as the means ± SDs. Asterisks indicate *p* values, *p < 0.05, **p < 0.01, ***p < 0.001, ****p < 0.0001, and ns represents no significant difference. n = 3 per group.

Actin‐tracker red rhodamine and TRAP staining were used to detect the inhibition effect of the PLG‐*g*‐TA/VEGF/Sr‐BGNPs hydrogel on RNAKL‐induced osteoblastic differentiation in vitro. As shown in **Figure** **5**A,B, osteoclasts appeared in the positive blank group after 5 days of 50 ng mL^−1^ RANKL induction and exhibited a large number of fused cells with several intracellular nuclei. The quantitative analysis showed that compared with that in the positive blank group, the RANKL‐induced osteoblastic differentiation of Raw264.7 cells was significantly inhibited by treatment with the PLG‐*g*‐TA/Sr‐BGNPs and PLG‐*g*‐TA/VEGF/Sr‐BGNPs hydrogels, as indicated by the decrease in the size and number of osteoblasts (Figure 5E,F) (*p* < 0.0001). Moreover, the difference between the two groups was not statistically significant (*p* > 0.05).

The results of toluidine blue staining of bovine bone slices are shown in Figure 5C. The negative blank group maintained the original texture structure of the bovine bone slices. In contrast, the texture of the bovine bone slices in the positive blank group was destroyed after 10 days of RANKL induction, with the appearance of purple dark‐stained rounded or subrounded resorption traps, and part of the bone surface was destroyed with irregular translucent voids on the edges. However, in the PLG‐*g*‐TA/Sr‐BGNPs and PLG‐*g*‐TA/VEGF/Sr‐BGNPs groups, although purple dark‐stained round resorption traps also appeared, the degree of bone destruction was less severe than that in the other positive groups, demonstrating that the composite hydrogels loaded with Sr‐BGNPs had a greater ability to inhibit the bone resorption of osteoclasts.

As shown in Figure 5G–J, the RT‒PCR results showed that the negative blank group expressed only a minimal amount of Trap (ACP5) (Figure 5G), NFATc1 (Figure 5H), c‐Fos (Figure 5I), and MMP9 (Figure 5J) mRNA after 5 days of RANKL induction, while the relative RNA expression of the above four genes in the positive blank group was significantly greater than that in the negative blank group (*p* < 0.0001). However, the relative RNA expression in the PLG‐*g*‐TA/Sr‐BGNPs and PLG‐*g*‐TA/VEGF/Sr‐BGNPs groups decreased significantly compared with that in the positive blank group (*p* < 0.0001), which showed that the composite hydrogels loaded with Sr‐BGNPs effectively suppressed the expression of genes related to osteoblast differentiation and maturation.

Taken together, the above results demonstrated that PLG‐*g*‐TA/VEGF/Sr‐BGNPs inhibited the osteoblastic differentiation and bone resorption of Raw264.7 cells induced by RANKL in vitro.

### 2.7 Evaluation of Bone Formation In Vivo

#### 2.7.1 Microcomputed Tomography (Micro‐CT)

**Figure 6:**
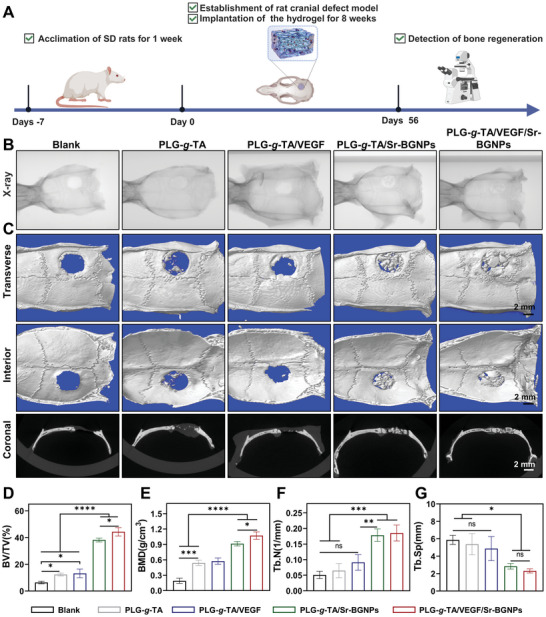
In vivo bone regeneration evaluation of different hydrogels in a rat calvarial defect model. A) Schematic illustration showing the design of calvarial defect treatment and treatment timeline. B) Representative images of X‐ray images of the rat cranial bone defect area at 8 weeks after the implantation of hydrogels. C) Representative 3D reconstructed images obtained from micro‐CT‐scanned calvarial bone after the implantation of hydrogels, including transverse, interior, and coronal sections (scale bar = 2 mm). D‐G) Quantitative analysis of new bone formation from micro‐CT results at 8 weeks after the implantation of hydrogels: BV/TV D), BMD E), Tb.N F), and Tb.Sp G). The data in Figure 6D–G were analyzed using one‐way ANOVA, and were presented as the means ± SDs. Asterisks indicate *p* values, *p < 0.05, **p < 0.01, ***p < 0.001, ****p < 0.0001, and ns represents no significant difference. *n* = 3 per group.

Given the enhanced osteogenic and angiogenic performance of the PLG‐*g*‐TA/VEGF/Sr‐BGNPs composite hydrogel in vitro, a 5 mm diameter SD rat cranial defect model was further created to evaluate the bone regeneration ability of the composite hydrogel in vivo (**Figure** **6**A). Eight weeks postoperatively, X‐ray and micro‐CT were first used to detect the efficacy of bone regeneration in the five groups. Figure 6B,C show the typical X‐ray and micro‐CT images of bone defects repaired in each group at 8 weeks. The amount of bone regeneration in the PLG‐*g*‐TA/VEGF/Sr‐BGNPs and PLG‐*g*‐TA/Sr‐BGNPs groups was significantly greater than that in the blank and PLG‐*g*‐TA groups at 8 weeks. In particular, the bone defects in the PLG‐*g*‐TA/VEGF/Sr‐BGNPs group were almost entirely occupied by newly formed bone. The repair ability of the defects in each group decreased in the order of PLG‐*g*‐TA/VEGF/Sr‐BGNPs > PLG‐*g*‐TA/Sr‐BGNPs > PLG‐*g*‐TA/VEGF > PLG‐*g*‐TA > blank.

Furthermore, the bone volume/tissue volume (BV/TV), bone mineral density (BMD), trabecular number (Tb.N), trabecular separation (Tb.Sp), and trabecular thickness (Tb.Th) were calculated to quantitatively analyze the regeneration of new bone at the cranial defect. Compared with the other four groups, the PLG‐*g*‐TA/VEGF/Sr‐BGNPs group exhibited the highest BV/TV (*p* < 0.05; Figure 6D). Similarly, the BMD of the new bone in the four hydrogel groups was greater than that in the blank group (Figure 6E). As mentioned in the SEM image of the hydrogel, the porous and ordered internal structure of the hydrogel filling the defect site provided good spatial support for cell recruitment, adhesion, and growth toward the inside of the defect site; thus, the new bone was of higher quality and had a larger BMD. Moreover, Sr‐BGNPs, in addition to achieving in situ mineralization, further promoted cell proliferation and osteogenic differentiation through the release of functional ions and promoted an increase in the BMD. Tb.N (Figure 6F), Tb.Sp (Figure 6G), and Tb.Th (Figure S21, Supporting Information) were further analyzed, and the results showed that the differences among the blank, PLG‐*g*‐TA, and PLG‐*g*‐TA/VEGF groups were not statistically significant (*p* > 0.05), whereas the differences among the PLG‐*g*‐TA/Sr‐BGNPs and PLG‐*g*‐TA/VEGF/Sr‐BGNPs groups were statistically significant compared with the blank and PLG‐*g*‐TA groups (*p* < 0.05). Moreover, compared with those of the blank group, the synergistic treatment of the PLG‐*g*‐TA/VEGF/Sr‐BGNPs group had the best results, with increased BV/TV, BMD, Tb.N, and Tb.Th and decreased Tb.Sp, reflecting a more compact structure of the newly formed bone, which can significantly resist external forces.^[^ ^45^ ^]^

#### 2.7.2 Histological Analysis

**Figure 7:**
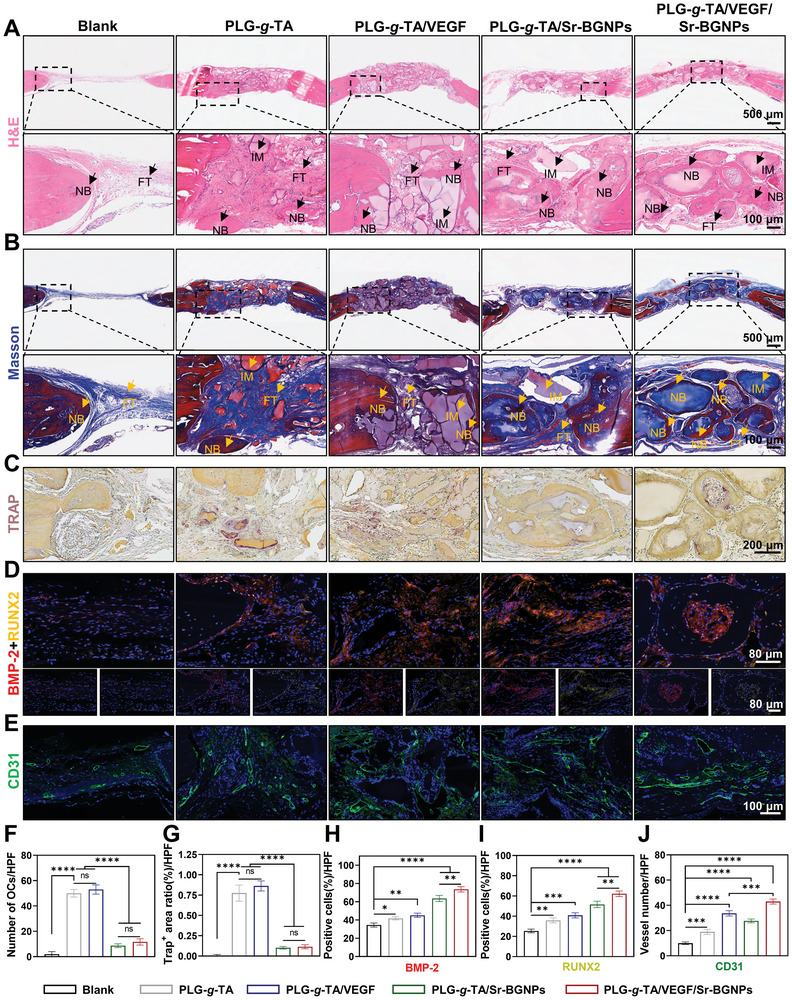
Bone formation evaluation in vivo. A,B) H&E (A) and Masson's trichrome (B) staining of new bone at 8 weeks after the implantation of hydrogels. The lower panel images of H&E and Masson (scale bar = 100 µm) are local high‐magnification images of the corresponding areas of the black boxes in the upper panel (scale bar = 500 µm, NB: new bone; IM: implanted material; FT: fibrous tissue). C) TRAP staining of the rat cranial bone defect area at 8 weeks after hydrogel implantation (scale bar = 200 µm); D) Immunofluorescence staining for protein markers of BMP‐2 and RUNX2. The blue fluorescence represents the nuclei of the cells, the red fluorescence represents the BMP‐2 protein expressed by the cells, and the yellow fluorescence represents the RUNX2 protein expressed by the cells in the repair area. E) Immunofluorescence staining for the protein marker CD31. The blue fluorescence represents the nuclei of the cells, and green fluorescence represents the CD31‐labeled blood vessels in the repair area. F) Quantitative analysis of the number of osteoclasts in the high‐magnification view field in each group (OCs, osteoclasts). G) Quantitative analysis of the percentage of Trap^+^ stained area in the high‐magnification view field of each group. H) Quantitative analysis of the average percentage of positive cells expressing the BMP‐2 protein marker in the high‐magnification view field of each group. I) Quantitative analysis of the average percentage of positive cells expressing the RUNX2 protein marker in the high‐magnification view field of each group. J) Quantitative analysis of the number of CD31‐labeled blood vessels with obvious lumen structures in the high‐magnification view field of each group. The data in Figure 7F–J were analyzed using one‐way ANOVA, and were presented as the means ± SDs. Asterisks indicate *p* values, *p < 0.05, **p < 0.01, ***p < 0.001, ****p < 0.0001, and ns represents no significant difference. *n* = 3 per group.

At 8 weeks after hydrogel implantation, H&E (**Figure** **7**A) and Masson's trichrome (Figure 7B) staining revealed that the bone defect area was filled with new bone tissue in the PLG‐*g*‐TA/Sr‐BGNPs and PLG‐*g*‐TA/VEGF/Sr‐BGNPs groups, while the center of the defect area in the blank group was filled with only a tiny amount of sparse fibrous tissue. The local high‐magnification view of the defect edge in the blank group showed a small amount of new bone with a few blood vessels included. Notably, in addition to the presence of many mature and intact new bones in the PLG‐*g*‐TA/VEGF/Sr‐BGNPs group, new bones were also observed at the edge of the undegraded hydrogel, and a small amount of hydrogel remained in the center of some mature bones. We could conclude that the new bones mainly originated from the edge of the hydrogel and progressively developed toward the center to replace the hydrogel until all became bone tissues as the hydrogel was degraded and new bones were formed. In addition, H&E staining of the heart, liver, spleen, lung, and kidney of the rats after 8 weeks of hydrogel implantation showed no systemic toxicity in vivo (Figure S22, Supporting Information), demonstrating that the PLG‐*g*‐TA/VEGF/Sr‐BGNPs hydrogel could be a safe therapeutic material suitable for bone regeneration.

Osteoclasts in the sections can be visualized by TRAP staining and appear under the microscope as large cells with dark red cytoplasm and varying numbers of nuclei. At 8 weeks post‐surgery, the results of TRAP staining in each group are shown in Figure 7C. There were very few TRAP‐positive (Trap^+^) osteoclasts at the edge of the new bone in the blank group, while osteoclasts were mostly distributed at the interface adjacent to the hydrogels and bone in each hydrogel group. The quantitative analysis confirmed that the PLG‐*g*‐TA and PLG‐*g*‐TA/VEGF groups had more Trap^+^ osteoclasts, and the difference between the two groups in terms of osteoclast number was not statistically significant (*p* > 0.05). However, the PLG‐*g*‐TA/Sr‐BGNPs and PLG‐*g*‐TA/VEGF/Sr‐BGNPs groups had significantly fewer Trap^+^ osteoclasts than the PLG‐*g*‐TA and PLG‐*g*‐TA/VEGF groups (*p* < 0.0001) (Figure 7F). Moreover, both the PLG‐*g*‐TA/Sr‐BGNPs and PLG‐*g*‐TA/VEGF/Sr‐BGNPs groups had significantly lower percentages of the Trap^+^ area than did the PLG‐*g*‐TA and PLG‐*g*‐TA/VEGF groups (*p* < 0.0001) (Figure 7G), demonstrating that the Sr‐BGNPs‐loaded composite hydrogel conferred strong osteoclastic inhibitory ability, which was consistent with the in vitro results.

Immunofluorescence staining for protein markers of BMP‐2 and RUNX2 is shown in Figure 7D. The quantitative analysis confirmed that the average percentage of positive cells expressing BMP‐2 and RUNX2 protein markers in hydrogel treatment groups was greater than that in the blank group (p < 0.05) (Figure 7H,I). Among them, the PLG‐g‐TA/VEGF/Sr‐BGNPs group had the highest percentage of positive cells, demonstrating that the PLG‐g‐TA/VEGF/Sr‐BGNPs composite hydrogel had the most powerful osteogenic ability. Moreover, immunofluorescence staining for the protein marker of platelet endothelial cell adhesion molecule‐1 (CD31) is shown in Figure 7E. As shown in Figure 7J, quantitative analysis also confirmed that the number of CD31‐labeled vessels with distinct luminal structures was greater in the hydrogel treatment groups than in the blank group (*p* < 0.05). Moreover, the average number of vessels in the PLG‐g‐TA/VEGF/Sr‐BGNPs group was the greatest, demonstrating that the PLG‐g‐TA/VEGF/Sr‐BGNPs composite hydrogel also had the most powerful angiogenic ability.

## 3 Conclusion

The inorganic‒organic multifunctional composite hydrogel (PLG‐*g*‐TA/VEGF/Sr‐BGNPs) loaded with Sr‐BGNPs and VEGF prepared in this study has good biosafety both in vitro and in vivo. The mechanical stiffness and the ability to induce mineralization of the hydrogel were significantly enhanced by the addition of Sr‐BGNPs. Moreover, the loading of VEGF effectively promoted the construction of vascularized tissue‐engineered bone grafts. The PLG‐*g*‐TA/VEGF/Sr‐BGNPs hydrogel can biomineralize in situ and slow the release of functional ions and factors to promote osteogenesis and angiogenesis. Moreover, the PLG‐*g*‐TA/VEGF/Sr‐BGNPs hydrogel synergistically accelerated the *in‐situ* regeneration of bone tissue at the defective site by promoting the proliferation, migration, and osteogenic differentiation of rBMSCs; promoting the differentiation of HUVECs to form blood vessels; and inhibiting the differentiation of osteoclasts and their bone resorption ability. In summary, this study proposes a new way of treating bone defects in the future, and PLG‐*g*‐TA/VEGF/Sr‐BGNPs hydrogel is also a promising biomaterial for artificial renewable bone grafts.

## 4 Experimental Section

Materials and methods, cell experiments, animal experiments, and other data are available in the Supporting Information. All experimental procedures involving animals were conducted with the approval of the Institutional Animal Ethics Committee of Sichuan University West China Hospital (approval number: 20 220 519 015).

### Ethics Approval Statement

All experimental procedures involving animals were conducted with the approval of the Institutional Animal Ethics Committee of Sichuan University West China Hospital (approval number: 20220519015). All the animal procedures used were based on the “Guide for the Care and Use of Laboratory Animals” of the National Research Council (US) (2011). Essential prevention measures were used to minimize the suffering of laboratory animals, and the total number of animals used in the study was strictly controlled.

## Conflict of Interest

The authors declare no conflict of interest.

## Author Contributions

C.H., S.S., and M.Q. contributed equally to this work. C.H., S.S., and MY.Q. designed the study, performed the experiments, and wrote the manuscript. X.R., ZC.D., XX.F., L.L., DP.W., and WN.Z. performed the experiments, analyzed the results, and revised the manuscript. ZY.L., YW.L., and ZK.Z. provided critical comments and revised the manuscript. All authors read and approved the final manuscript.

## Supporting information

Supporting Information

## Data Availability

The data that support the findings of this study are available in the supplementary material of this article.

## References

[1] a) P. Yu , F. Yu , J. Xiang , K. Zhou , L. Zhou , Z. Zhang , X. Rong , Z. Ding , J. Wu , W. Li , Z. Zhou , L. Ye , W. Yang , Adv. Mater. 2022, 34, 2107922; 10.1002/adma.202107922 34837252

[2] M. K. Sen , T. Miclau , Injury 2007, 38 Suppl 1, S75. 17383488 10.1016/j.injury.2007.02.012

[3] Y. Fillingham , J. Jacobs , Bone Joint J 2016, 98‐B, 6. 10.1302/0301-620X.98B.36350 26733632

[4] X. Xue , Y. Hu , Y. Deng , J. Su , Adv. Funct. Mater. 2021, 31, 2009432.

[5] a) A. S. Hoffman , Adv Drug Deliv Rev 2002, 54, 3;11755703

[6] Q. Wang , J. L. Mynar , M. Yoshida , E. Lee , M. Lee , K. Okuro , K. Kinbara , T. Aida , Nature 2010, 463, 339. 20090750 10.1038/nature08693

[7] C. Lei , J. H. Song , S. Li , Y. N. Zhu , M. Y. Liu , M. C. Wan , Z. Mu , F. R. Tay , L. N. Niu , Biomaterials 2023, 296, 122066. 36842238 10.1016/j.biomaterials.2023.122066

[8] M. Sun , X. Sun , Z. Wang , S. Guo , G. Yu , H. Yang , Polymers (Basel) 2018, 10, 1290. 30961215 10.3390/polym10111290 PMC6401825

[9] G. S. Krishnakumar , S. Sampath , S. Muthusamy , M. A. John , Mater Sci Eng C Mater Biol Appl 2019, 96, 941. 30606606 10.1016/j.msec.2018.11.081

[10] E. Zeimaran , S. Pourshahrestani , A. Fathi , N. Razak , N. A. Kadri , A. Sheikhi , F. Baino , Acta Biomater. 2021, 136, 1. 34562661 10.1016/j.actbio.2021.09.034

[11] R. Fujisawa , Y. Wada , Y. Nodasaka , Y. Kuboki , Biochim. Biophys. Acta 1996, 1292, 53. 8547349 10.1016/0167-4838(95)00190-5

[12] a) X. Guan , M. Avci‐Adali , E. Alarcin , H. Cheng , S. S. Kashaf , Y. Li , A. Chawla , H. L. Jang , A. Khademhosseini , Biotechnol. J. 2017, 12, 1600394; 10.1002/biot.201600394 PMC5503693 28220995

[13] X. Xue , Y. Hu , Y. Deng , J. Su , Adv. Funct. Mater. 2021, 31, 2009432.

[14] a) X. Ding , J. Shi , J. Wei , Y. Li , X. Wu , Y. Zhang , X. Jiang , X. Zhang , H. Lai , Sci. Adv. 2021, 7, eabj7857; 34890238 10.1126/sciadv.abj7857 PMC8664252

[15] a) P. Sepulveda , J. R. Jones , L. L. Hench , J Biomed Mater Res 2002, 59, 340; 11745571 10.1002/jbm.1250

[16] C. Gao , Y. Li , X. Wang , Materials Research and Application 2009, 3, 112.

[17] a) E. Bonnelye , A. Chabadel , F. Saltel , P. Jurdic , Bone 2008, 42, 129; 17945546 10.1016/j.bone.2007.08.043

[18] a) Y. Zhu , Y. Ouyang , Y. Chang , C. Luo , J. Xu , C. Zhang , W. Huang , Mol. Med. Rep. 2013, 7, 1129; 23446964 10.3892/mmr.2013.1341

[19] Y. Zha , T. Lin , Y. Li , X. Zhang , Z. Wang , Z. Li , Y. Ye , B. Wang , S. Zhang , J. Wang , Biomaterials 2020, 247, 119985. 32272301 10.1016/j.biomaterials.2020.119985

[20] D. Kaigler , Z. Wang , K. Horger , D. J. Mooney , P. H. Krebsbach , J. Bone Miner. Res. 2006, 21, 735. 16734388 10.1359/jbmr.060120

[21] A. Tavakolizadeh , M. Ahmadian , M. H. Fathi , A. Doostmohammadi , E. Seyedjafari , A. Ardeshirylajimi , ASAIO J. 2017, 63, 512. 28033183 10.1097/MAT.0000000000000509

[22] T. Kokubo , H. Takadama , Biomaterials 2006, 27, 2907. 16448693 10.1016/j.biomaterials.2006.01.017

[23] B. Lei , X. Chen , Y. Wang , N. Zhao , C. Du , L. Fang , Biomed. Mater. 2010, 5, 054103. 20876955 10.1088/1748-6041/5/5/054103

[24] a) L. L. Hench , J. Wilson , Science 1984, 226, 630; 6093253 10.1126/science.6093253

[25] C. He , C. Zhao , X. Guo , Z. Guo , X. Chen , X. Zhuang , S. Liu , X. Jing , Journal of Polymer Science Part A: Polym. Chem. 2008, 46, 4140.

[26] a) F. Lee , J. E. Chung , M. Kurisawa , Soft Matter 2008, 4, 880; 32907194 10.1039/b719557e

[27] B. Jeong , Y. H. Bae , S. W. Kim , Macromolecules 1999, 32, 7064.

[28] Timothy, A. , J. Nutr. 2008, 138, 415S. 18203913 10.1093/jn/138.2.415S

[29] M. Harada , N. Udagawa , K. Fukasawa , B. Y. Hiraoka , M. Mogi , J Dent Res 1986, 65, 125. 3003174 10.1177/00220345860650020601

[30] S. Li , L. Zhang , C. Liu , J. Kim , K. Su , T. Chen , L. Zhao , X. Lu , H. Zhang , Y. Cui , X. Cui , F. Yuan , H. Pan , Bioact Mater 2023, 23, 101. 36406252 10.1016/j.bioactmat.2022.10.021 PMC9664355

[31] W. Liu , X. Dan , W. W. Lu , X. Zhao , C. Ruan , T. Wang , X. Cui , X. Zhai , Y. Ma , D. Wang , W. Huang , H. Pan , ACS Appl. Mater. Interfaces 2019, 11, 9557. 30720276 10.1021/acsami.8b20580

[32] a) R. Subbiah , M. A. Ruehle , B. S. Klosterhoff , A. S. P. Lin , M. H. Hettiaratchi , N. J. Willett , L. E. Bertassoni , A. J. Garcia , R. E. Guldberg , Acta Biomater. 2021, 127, 180; 33823326 10.1016/j.actbio.2021.03.066

[33] Q. Min , X. Yu , J. Liu , Y. Zhang , Y. Wan , J. Wu , Pharmaceutics 2020, 12, 574. 32575684 10.3390/pharmaceutics12060574 PMC7355909

[34] S. Hafeez , A. A. Aldana , H. Duimel , F. A. A. Ruiter , M. C. Decarli , V. Lapointe , C. van Blitterswijk , L. Moroni , M. B. Baker , Adv. Mater. 2023, 35, 2207053. 10.1002/adma.202207053 36858040

[35] a) T. Ding , J. Li , X. Zhang , L. Du , Y. Li , D. Li , B. Kong , S. Ge , Biomater. Sci. 2020, 8, 2459; 32191780 10.1039/d0bm00102c

[36] V. Midy , J. Plouet , Biochem. Biophys. Res. Commun. 1994, 199, 380. 8123039 10.1006/bbrc.1994.1240

[37] A. D. Berendsen , B. R. Olsen , J. Intern. Med. 2015, 277, 674. 25779338 10.1111/joim.12364

[38] H. Eckardt , M. Ding , M. Lind , E. S. Hansen , K. S. Christensen , I. Hvid , J Bone Joint Surg Br 2005, 87, 1434. 16189323 10.1302/0301-620X.87B10.16226

[39] M. Huang , R. G. Hill , S. C. Rawlinson , Acta Biomater. 2016, 38, 201. 27131573 10.1016/j.actbio.2016.04.037

[40] P. J. Marie , Bone 2007, 40, S5.

[41] F. Yang , D. Yang , J. Tu , Q. Zheng , L. Cai , L. Wang , Stem Cells 2011, 29, 981. 21563277 10.1002/stem.646

[42] D. Huang , F. Zhao , W. Gao , X. Chen , Z. Guo , W. Zhang , Regen Biomater 2020, 7, 303. 32523732 10.1093/rb/rbaa004 PMC7266663

[43] X. Ding , X. Li , C. Li , M. Qi , Z. Zhang , X. Sun , L. Wang , Y. Zhou , ACS Biomater. Sci. Eng. 2019, 5, 4574. 33448831 10.1021/acsbiomaterials.9b00584

[44] G. Hong , Z. Chen , X. Han , L. Zhou , F. Pang , R. Wu , Y. Shen , X. He , Z. Hong , Z. Li , W. He , Q. Wei , Clin. Transl. Med. 2021, 11, e392. 34047464 10.1002/ctm2.392 PMC8140192

[45] H. S. Moon , Y. Y. Won , K. D. Kim , A. Ruprecht , H. J. Kim , H. K. Kook , M. K. Chung , Surg Radiol Anat 2004, 26, 466. 15146293 10.1007/s00276-004-0247-x

