# Supplementary material for: A Composite Hydrogel Functionalized by Borosilicate Bioactive Glasses and VEGF for Critical‐Size Bone Regeneration
Source: Adv Sci (Weinh). 2024 May 7;11(26):2400349. doi: 10.1002/advs.202400349 (PMC11234436; doi:10.1002/advs.202400349)
Supplement: Supplementary file 1 — Supporting Information [file ADVS-11-2400349-s001.pdf]

ADVANCED SCIENCE
Open Access

Supporting Information

for *Adv. Sci.*, DOI 10.1002/advs.202400349

A Composite Hydrogel Functionalized by Borosilicate Bioactive Glasses and VEGF for Critical-Size Bone Regeneration

Chao Huang, Shun Shi, Muyan Qin, Xiao Rong, Zichuan Ding, Xiaoxue Fu, Weinan Zeng, Lei Luo, Deping Wang, Zeyu Luo, Yiwen Li* and Zongke Zhou**

Supplementary Information

A Composite Hydrogel Functionalized by Borosilicate Bioactive Glasses and VEGF for Critical-size Bone Regeneration

4. Experimental Methods

4.1 Materials

Tetraethoxysilane (TEOS), tributyl borate (TBB), calcium nitrate tetrahydrate ($Ca(NO_3)_2 \cdot 4H_2O$), strontium nitrate ($Sr(NO_3)_2$), cetyltrimethylammonium bromide (CTAB), and hydrogen peroxide (H_2O_2) were obtained from Sigma Aldrich (Shanghai, China). L-glutamic acid γ-benzyl ester (BLG), triphosgene, 33 wt.% hydrobromic acid (HBr) in acetic acid, triethylamine, and horseradish peroxidase (HRP) were purchased from Aladdin Biochemical Technology (Shanghai, China). N,N-dimethylformamide, n-hexylamine, N-hydroxysuccinimide (NHS), and 1-(3-dimethylaminopropyl)-3-ethylcarbodiimide hydrochloride (EDC·HCl) were purchased from Energy Chemical (Anhui, China). Simulated body fluid (SBF, pH=7.4) was purchased from Phygene (Fujian, China). Human $VEGF^{165}$ ($hVEGF^{165}$) protein was obtained from ACROBiosystems (Beijing, China). Phosphate-buffered saline (PBS, pH 7.4, 0.01 M) solution was prepared in the laboratory. Unless otherwise mentioned, all the measurements were performed at room temperature.

4.2 Instrumentation

Scanning electron microscopy (SEM) and energy dispersive spectroscopy (EDS) imaging were conducted on a Nova SEM450 (FEI, USA). Transmission electron microscopy (TEM) imaging was conducted on a Tecnai G2 F20 S-TWIN (FEI, USA) at 200 keV. Fourier transform infrared spectroscopy (FTIR) was conducted on a SPECTRUM ONE Nicolet (PE, USA). X-ray diffraction (XRD) was conducted by EMPYREAN (PANALYTICAL, Netherlands). ^1H nuclear magnetic resonance (^1H NMR) spectra were measured by a Bruker AV II-400 MHz (Bruker, Switzerland). A dynamic light scattering (DLS) particle size analyzer (Brookhaven, USA) was used to determine the diameters of the particles. The concentrations of silicon (Si), calcium (Ca), boron (B), and strontium (Sr) were detected by inductively coupled plasma–optical emission spectrometry (ICP–OES) (Perkin Elmer, Optima 8000, USA). The pH of the solution was obtained by an acidity meter S220-K (Mettler Toledo, Switzerland). The mechanical properties of the hydrogels were measured on a dynamic mechanical analysis rheometer (Anton Paar MCR 302, Austria). Ultrapure water (18.2 MΩ cm^{-1} at 25 °C) was

purified by a Milli-Q system (Milli-Q® IQ 7000, Merck, Germany). All the measurements were performed at room temperature if not specially mentioned.

4.3 Synthesis of strontium-doped borosilicate bioactive glass nanoparticles (Sr-BGNPs)

The Sr-BGNPs were synthesized in a multicomponent 70SiO_2-10B_2O_3-14CaO-6SrO system using a sol-gel method. The appropriate molar ratios of TEOS, TBB, $Ca(NO_3)_2·4H_2O$, and $Sr(NO_3)_2$ were defined according to the molar ratios of SiO_2, B_2O_3, CaO, and SrO. Briefly, 200 mL of deionized water was added to a flat-bottomed flask and placed in a constant-temperature water bath at 40 °C with magnetic stirring. After the temperature stabilized, CTAB (0.01 mol/L) was added, and then 3.3 mL of concentrated ammonia (28%, 15 mol/L) was added, resulting in an ammonia concentration of 0.25 mol/L. Then, 2.8 mL of TEOS and 1 mL of TBB were mixed and added dropwise to the bottle, and stirring was continued for 30 minutes (min). Then, 0.588 g of $Ca(NO_3)_2·4H_2O$ and 0.266 g of $Sr(NO_3)_2$ were dissolved separately in 5 mL of deionized water, added dropwise to the bottle, and stirred for 4 hours (h). The solution was then transferred to a centrifuge tube and centrifuged at 4000 rpm for 5 min, after which the supernatant was discarded. Anhydrous ethanol and deionized water were added to clean the product, and the product was placed in a -20 ℃ refrigerator overnight and then freeze-dried in a lyophilizer for 24 h. Finally, the product was heat-treated at 600 ℃ for 3 h at 20 ℃/min in air.

4.4 Characterization of Sr-BGNPs

4.4.1 Microscopic observations

The shape and surface morphology of the Sr-BGNPs were evaluated using SEM. The samples were coated with a gold layer before microscopy. Moreover, the surface of the Sr-BGNPs was scanned by EDS to analyze the elemental composition of the surface and the distribution of Si, Ca, B, Sr, and oxygen (O). The sample was prepared by dispersing a small amount of Sr-BGNPs powder in anhydrous ethanol and homogeneously dispersed under ultrasonic waves. Then, the suspension was dropped on 230 mesh copper TEM grids covered with thin amorphous carbon films. TEM was performed after the ethanol evaporated cleanly.

4.4.2 Dynamic light scattering (DLS) and zeta potential

Sr-BGNPs were added to deionized water, sonicated for 10 min, and then transferred to a clean cuvette. The particle size and surface charge were detected by DLS and a zeta potential tester, and the measurements were repeated in triplicate.

4.4.3 FTIR and XRD

The functional groups of the Sr-BGNPs were analyzed using FTIR. The Sr-BGNPs and KBr were mixed at a mass ratio of 1:100, and the FTIR spectra were recorded from 400 to 4400 cm^{-1} with a resolution of 4 cm^{-1}. The crystal structure of the Sr-BGNPs was determined by XRD (with Cu-Kα radiation, a scanning speed of 2°/min, a scanning range of 10-80°, and a scanning diffraction angle of 2θ).

4.4.4 Ionic release and apatite formation ability

Sr-BGNPs were immersed in SBF at 1 g/200 mL. Briefly, 50 mg of Sr-BGNPs was added to a 15 mL centrifuge tube, followed by the addition of 10 mL of SBF, and the mixture was placed on a shaking table at 37 °C and 100 rpm. The time was recorded as 0 d, and three groups of parallel samples were set up. At 0.25, 0.5, 1, 2, 4, 6, 8, 12, 16, 20, 24, and 28 days of immersion, 3 mL of the supernatant was removed to test the pH of the solution and the concentrations of Si, Ca, B, and Sr ions using a pH meter and an ICP–OES, respectively. Meanwhile, 3 mL of fresh SBF was added to keep the liquid in the tube at 10 mL. Moreover, the Sr-BGNPs were removed after 1, 3, 7, and 14 days of immersion, rinsed with deionized water and acetone, and dried in an electrically heated oven. The resulting samples were subjected to FTIR, XRD, SEM, and EDS to detect the formation of apatite.

4.5 Preparation and characterization of the PLG-*g*-TA copolymer

Thirty grams of BLG and 18.8 g of triphosgene were suspended in 300 mL of anhydrous tetrahydrofuran with magnetic stirring at 50 ℃ for 30 min until the mixture turned into a transparent solution. The solution was poured into 2000 mL of iced petroleum ether for sedimentation and filtered to leave the product. The product was dissolved by adding ethyl acetate and transferred to a separatory funnel, and saturated sodium bicarbonate and saturated sodium chloride solution were added successively. The lower layer of liquid was discarded after being inverted and mixed, while the rest of the solution was collected using a flat-bottomed conical flask, to which anhydrous magnesium sulfate was added, and then it was sealed and placed at -20 ℃ overnight. The magnesium sulfate was filtered the next day, and the filtrate was extracted under vacuum for 6 h to obtain BLG-NCA.

PBLG was synthesized by the ring-opening polymerization of BLG-NCA using triethylamine as the initiator. BLG-NCA (22.127 g) was suspended in 210 mL of anhydrous N,N-dimethylformamide under a nitrogen flow. Fifty-five microlitres of n-hexylamine was added when the BLG-NCA was dissolved, and the reaction was carried out for 3 d. Then, the solution was poured into a beaker containing 2000 mL of ice-cold anhydrous ether for sedimentation. After filtration, the product was collected, and a small amount of anhydrous

ether was added. The mixture was then sealed and placed at 4 °C overnight. The next day, the PBLG was obtained by vacuum filtration.

PLG was obtained by PBLG deprotection. Briefly, 16.84 g of PBLG was dissolved in 51 mL of dichloroacetic acid, and 17 mL of 33 wt% HBr solution in acetic acid was added to the reaction mixture. Two hours later, the product was settled in ice-cold anhydrous ether, filtered, and dissolved in an appropriate amount of DMSO. Then, the product was transferred to a dialysis bag (3500 Da), purified by dialysis against deionized water for 3 d and collected by lyophilization for 3 d.

A poly(L-glutamic acid)-graft-tyramine (PLG-*g*-TA) copolymer was prepared by coupling PLG with TA via an EDC/NHS-activated amidation reaction. Briefly, 1 g of PLG was first dissolved in 20 mL of DMSO and activated using 0.089 g of NHS and 0.1485 g of EDC·HCl. The solution was allowed to react for 3 h. TA (0.106 g) was then added to the solution and stirred for 24 h. The product was transferred to a dialysis bag (3500 Da) and purified by dialysis against deionized water for 3 d. The solution was adjusted to neutral pH (7.40) with sodium bicarbonate to make the product soluble in water. The final product, the PLG-*g*-TA copolymer, was collected by lyophilization for 3 d.

Moreover, the ^{1}H NMR spectra of BLG-NCA, PBLG, PLG, and the PLG-*g*-TA copolymer were measured. BLG-NCA and PBLG were solubilized using CF_3COOD, while PLG and PLG-*g*-TA were solubilized using D_2O.

4.6 Synthesis of the PLG-*g*-TA/VEGF/Sr-BGNPs hydrogel

The hydrogels were prepared via enzyme-catalyzed cross-linking in the presence of HRP and H_2O_2 in PBS with a polymer:HRP:H_2O_2 volume ratio of 2:1:1. Sol-gel tests were performed by the vial inversion method. If the mixed liquid in the vial did not flow within 30 s after inverting the vial, the mixture had transformed from the solution to the gel state, and the time from the addition of H_2O_2 to the formation of the gel was recorded as the gel formation time. According to the methods of Li *et al.* [1], 10% (w/v) PLG-*g*-TA copolymer solution was prepared using PBS containing 0 or 5 μg/mL hVEGF^{165}, and HRP (8 units/mL) and H_2O_2 solution (molar ratio of H_2O_2:TA = 0.4) were added successively and vortex mixed to obtain PLG-*g*-TA and PLG-*g*-TA/VEGF hydrogels. Sr-BGNPs were added to the PLG-*g*-TA copolymer solution at 5% (w/v) of the total formed hydrogel volume to obtain the PLG-*g*-TA/Sr-BGNPs and PLG-*g*-TA/VEGF/Sr-BGNPs hydrogels.

4.7 Characterization of the PLG-*g*-TA/VEGF/Sr-BGNPs hydrogels

4.7.1 Morphology of the hydrogel

First, 120 μL of 10% (w/v) PLG-*g*-TA polymer, 60 μL of 8 units/mL HRP, and 60 μL of H_2O_2 (molar ratio of H_2O_2: TA = 0.4) solution were quickly mixed. Then, 200 μL of mixed solution was quickly transferred to a pipette with a diameter of 3 mm. Thirty minutes later, the sample was put into the liquid nitrogen to freeze solid and dried using a lyophilizer. Then, the samples placed in liquid nitrogen again to ensure that they were brittle and were sputter-coated with gold. The morphology of the hydrogel was observed using SEM.

4.7.2 Composition of the hydrogel

The PLG-*g*-TA and PLG-*g*-TA/Sr-BGNPs hydrogels were dried using a lyophilizer, and then FTIR was performed with the Sr-BGNPs. The results of the three samples were compared and analyzed.

4.7.3 Rheological properties of the hydrogels

The mechanical properties of the PLG-*g*-TA and PLG-*g*-TA/Sr-BGNPs hydrogels were measured on an Anton Paar MCR 302. An 8 mm diameter parallel plate was used. The test temperature was set at 25 °C, the test mode was oscillation mode, and the gap was set at 0.5 mm. Then, 40 μL of the PLG-*g*-TA polymer solution, 20 μL of the HRP solution, and 20 μL of the H_2O_2 solution were homogeneously mixed and immediately placed on the plate of the rheometer. The edges of the sample were cleanly wiped after setting the gap at 0.5 mm and sealed with silicone oil to prevent the moisture from volatilizing and affecting the results. The time sweep test was carried out during the crosslinking process of the hydrogels at a strain (γ) of 1%, a frequency (f) of 1 Hz, and an FN of 0 N to record the storage (G') and loss (G") moduli of the hydrogels.

As shown in **Figure S8**, the G' of the hydrogels increased quickly at the beginning and then reached a plateau, demonstrating the completion of the crosslinking reaction. Notably, a higher HRP concentration **(Figure S8A)** or copolymer concentration **(Figure S8B)** resulted in a markedly greater G' with a shorter gel formation time, which indicated that a higher polymer concentration favored the formation of hydrogels with high crosslinking density, thus providing higher G'. In contrast, a higher H_2O_2 concentration **(Figure S8C)** led to a higher G' with a prolonged gel formation time. The choice of H_2O_2 was determined according to live/dead cell staining. The results are shown in **Figure S9.** The number of the dead cells in the hydrogel group with a molar ratio of H_2O_2 to TA (nH_2O_2/nTA) of 0.5 was greater than that in 0.3 and 0.4 groups, which was considered to be related to the presence of excess H_2O_2 in the hydrogel which was not involved in the reaction in addition to the occurrence of normal apoptosis. The intermolecular reaction was not 100% consumed by H_2O_2, and there was also H_2O_2 that did not

participate in the reaction, which affected cell survival; therefore, subsequent experiments were carried out using a hydrogel group with an nH_2O_2/nTA ratio of 0.4. Thus, we chose final concentration of 5% (w/v) for the PLG-*g*-TA copolymer, an HRP concentration of 8 units/mL, and an nH_2O_2/nTA ratio of 0.4 for subsequent studies.

The effect of loading Sr-BGNPs on the G' and G" of the PLG-*g*-TA/Sr-BGNPs hydrogel was further evaluated by testing the time sweep, shear strain, and frequency scanning rheology of the hydrogel after the addition of 0%, 1%, 3%, 5%, 8%, and 10% (w/v) Sr-BGNPs, respectively. The time sweep test was performed as previously described. For the shear strain test, f was set to 1 Hz and γ ranged from 0.1-100%. For the frequency scanning test, γ was set to 1% and f ranged from 0.1-200 Hz.

4.7.4 Degradation of the hydrogel

For the *in vitro* degradation test, clean microcentrifuge tubes were weighed (*We*). A total of 400 µL hydrogel in microcentrifuge tubes was prepared as described before, and the resulting sample was accurately weighed as *W0*. Subsequently, PBS containing elastase (2 mg/mL) was added, and the samples were sealed and placed in an incubator at 37 °C. The microcentrifuge tubes were removed at the set time points, and the supernatant on the hydrogel was dried using filter paper, accurately weighed and recorded as *Wt*. At the same time, the PBS was replaced with freshly prepared elastase (2 mg/mL) to maintain enzyme activity. The percentage of remaining hydrogel mass at time point t was defined as *(Wt-We)/(W0-We)×100%*. The experiments were performed in triplicate.

For the *in vivo* degradation and biocompatibility tests, 6 Sprague–Dawley (SD) rats (200 to 250 g, 8 weeks old) were chosen. The dorsal subcutis of both sides was subcutaneously injected with 200 µL of mixed hydrogel precursor solution containing the PLG-*g*-TA copolymer, HRP, and H_2O_2. The rats were euthanized by inhalation of excess isoflurane at 30 min and at the 1st, 2^{dn}, 4th, 6th, and 8th weeks after injection. The hydrogel status was observed and photographed for recording. At the same time, the skin tissues surrounding the injection sites were surgically removed and stored in 10% neutral formaldehyde solution for fixation and then subjected to hematoxylin and eosin (H&E) staining.

4.7.5 Ionic release and apatite formation ability of the hydrogel

The PLG-*g*-TA/Sr-BGNPs and PLG-*g*-TA/VEGF/Sr-BGNPs hydrogels were immersed in SBF according to the ratio of the mass of the Sr-BGNPs to the volume of the SBF (1 g/200 mL). Subsequent experiments were performed as described above "4.4.4 Ionic release and apatite formation ability of Sr-BGNPs". Moreover, the PLG-*g*-TA/Sr-BGNPs and PLG-*g*-TA/VEGF/Sr-BGNPs hydrogels were immersed in SBF and replaced every 2 days. On the 7th

day, the hydrogels were removed, rinsed with deionized water, and then dried using a lyophilizer. The surface microtopography and apatite mineralization were characterized by SEM.

4.8 *In vitro* release of VEGF from the hydrogel

A total volume of 200 μL of the composite hydrogels PLG-*g*-TA/VEGF or PLG-*g*-TA/VEGF/Sr-BGNPs was soaked in 2 mL of PBS and placed in a constant temperature shaker at 37 °C and 100 rpm, and the supernatants were collected on the 2nd, 4th, 6th, 10th, 14th, 18th, 23rd, and 28th days, after while new 2 mL of PBS was added. The supernatants collected at each time point were stored at -80 °C. The experiments were performed in triplicate. After collection on the 28th day, 2 mL of PBS containing elastase at a concentration of 5 mg/mL was added. The supernatant was collected after complete degradation of the hydrogel, which was defined as the amount of VEGF that had not yet been released from the hydrogel on the 28th day. The optical density (OD) values of all the samples were measured at 450 nm using a QuantiCyto® human VEGF enzyme-linked immunosorbent assay (ELISA) kit. The standard curve of VEGF was then plotted using GraphPad Prism 9, and the regression equation of the standard curve was obtained. The percentage release of VEGF from the composite hydrogel PLG-*g*-TA/VEGF and PLG-*g*-TA/VEGF/Sr-BGNPs groups at different time points was calculated. The total amount of VEGF initially contained in the hydrogel was obtained by summing all released and unreleased VEGF on the 28th day.

4.9 *In vitro* experiments

4.9.1 Culture of cells

Bone marrow-derived mesenchymal stem cells (BMSCs) were isolated from the tibial bone marrow of SD rats (8-10 days). Briefly, after the rats were sacrificed, the tibias, knees, and ankle joints of the rats were removed, and the metaphysis was removed at both ends. Low-glucose Dulbecco's modified Eagle's medium (DMEM) containing 10% fetal bovine serum (FBS) and 1% penicillin–streptomycin was aspirated using a 1 mL syringe. A needle was inserted into the bone marrow cavity at one end. The medium was slowly pushed into the bone marrow cavity for rinsing, and the rinsing solution was transferred to a petri dish for culture. The cells were cultivated and passed from the P0 generation to the P3 generation, after which they were used for subsequent experiments. Flow cytometry was used to identify cell surface markers on the rBMSCs. CD29 and CD90 were selected as the stem cell surface markers, while CD34 and CD45 were selected as the hematopoietic cell surface markers. Cells that were

positive for CD29 and CD90 but negative for CD34 and CD45 could be identified as BMSCs. Moreover, the ability of the cells to induce osteogenic, chondrogenic, and lipogenic differentiation was also considered to be unique to stem cells, and the cells extracted in this study could be identified as rBMSCs.

A murine macrophage line (RAW264.7 cell) and human umbilical vein endothelial cells (HUVECs) were obtained from the Shanghai Institute of Cells, Chinese Academy of Sciences. RAW264.7 cells were cultured in minimum essential medium alpha (MEM α) basic (1X) (Gibco) supplemented with 10% FBS and 1% penicillin–streptomycin, while HUVECs were cultured in DMEM/F-12 (1:1) supplemented with 10% FBS and 1% penicillin–streptomycin. All the cells were incubated in a cell incubator at 37 °C, 5% CO_2, and 95% humidity.

4.9.2 Evaluation of biocompatibility in vitro

4.9.2.1 Cytocompatibility of Sr-BGNPs

TEM was used to observe whether the rBMSCs ingested monodisperse Sr-BGNPs and whether the uptake affected the cellular submicroscopic structure [2]. RBMSCs were plated into a 6-well plate at a density of 2×10^5/well overnight. The next day, an appropriate amount of Sr-BGNPs was weighed. Complete DMEM (10% FBS, 1% penicillin–streptomycin) was used to formulate conditioned medium supplemented with 250 μg/mL Sr-BGNPs. The medium of the positive control group was replaced with DMEM conditioned medium containing Sr-BGNPs, while that of the control group was replaced with complete DMEM without Sr-BGNPs. After 24 h, the cells were digested with trypsin, and the reaction was terminated by centrifugation to obtain cell pellets. Then, 1.2 mL of fixative containing 0.2 mL of 3% glutaraldehyde and 1 mL of PBS were added to the cell pellets, which were resuspended, placed at 4 °C for 5 min, and transferred to a 1.5 mL microcentrifuge tube. The cell pellets were obtained via centrifugation at 12,000 rpm for 10 min and further fixed with 3% glutaraldehyde and 1% osmium tetroxide, followed by dehydration in a series of graded acetone solutions (30%, 50%, 70%, 80%, 90%, 95%, 100%, 100%, and 100%). After dehydration, the cells were embedded in dehydrating agent and Epon812 embedding agent at 3:1, 1:1, and 1:3 ratios and finally embedded in Epon812. Eighty nanometer sections were obtained using a Leica Ultracut UCT ultramicrotome and collected on copper TEM grids. The cells were stained with dioxin acetate for 15 min and then with lead citrate staining solution for 2 min at 25 °C. Finally, TEM analysis was carried out.

Furthermore, a hemolysis test was performed to determine the hemocompatibility of the Sr-BGNPs with red blood cells. Briefly, solutions containing Sr-BGNPs at concentrations of 2000, 1000, 500, 250, 125, and 62.5 μg/mL were prepared with PBS. Briefly, 960 μL Sr-BGNPs

solutions of the above concentrations, PBS (negative group), and deionized water (positive group) were added to 1.5 mL microcentrifuge tubes. Then, 40 μL of rat blood was added and mixed gently. The tubes were then placed in a 37 °C incubator for 2 h. The experiments were performed in triplicate. After incubation, the samples were centrifuged at 1000 rpm for 10 min, and we observed whether obvious hemolysis occurred compared with that in the positive control group. One hundred microlitres of supernatant from each group was aspirated and transferred to a 96-well plate, and the absorbance of the supernatant at 545 nm was measured using a microplate reader. The calculation method was as follows: hemolytic ratio (HR) % = (OD sample - OD negative control)/(OD positive control - OD negative control) × 100%. Here, the OD sample represents the absorbance of the Sr-BGNPs tested at each concentration, the OD negative control represents the absorbance of the negative control group, and the OD positive control represents the absorbance of the positive control group.

4.9.2.2 Cytocompatibility of the PLG-g-TA copolymer and hydrogel

RBMSCs were plated into a 96-well plate at a density of 1×10^4/well overnight. The next day, solutions containing the PLG-*g*-TA copolymer at concentrations of 2000, 1500, 1000, 500, and 250 μg/mL were prepared with complete DMEM (10% FBS, 1% penicillin–streptomycin). Then, 100 μL of copolymer solution was added to the corresponding groups. An equal volume of medium without polymer was added to the blank group to obtain final concentrations of 1000, 750, 500, 250, 125, and 0 μg/mL copolymer solution. The incubation was continued for 24, 48, and 72 h. At each time point, the cells were washed with PBS twice. Subsequently, 110 μL of fresh medium containing 10 μL of cell counting kit 8 (CCK-8) solution was added, and after incubation for 2 h in the dark, 100 μL of supernatant from each well of each group was pipetted into a new 96-well plate. The OD value of each well at 450 nm was measured using a microplate reader.

Live/dead staining was employed to evaluate the cytotoxicity of the PLG-*g*-TA hydrogel. Briefly, a rBMSCs suspension of 1×10^6 cells/mL was made with complete DMEM, and the PLG-*g*-TA polymer was used to prepare a cell suspension with a 10% (w/v) polymer concentration. Then, 60 μL of cell suspension containing 10% (w/v) PLG-*g*-TA copolymer, 30 μL of HRP solution (8 units/mL), and 30 μL of H_2O_2 solution (nH_2O_2/TA=0.3, 0.4, and 0.5) were added to 1.5 mL microcentrifuge tubes and mixed rapidly. One hundred microlitres of the mixture was transferred to the center of a 15 mm diameter confocal dish to make a uniform dispersion, after which the PLG-*g*-TA hydrogel was formed. The experiments were performed in triplicate. Then, 2 mL of fresh medium was added to the dish, and the incubation was continued for 24 h. Subsequently, the live/dead staining solution was added to the dish, and the

mixture was incubated in the dark for 30 min. Finally, the cells were examined under a fluorescence microscope. Live cells were stained green, while dead cells were stained red.

4.9.3 Cell attachment

Two hundred microliters of the PLG-*g*-TA, PLG-*g*-TA/VEGF, PLG-*g*-TA/Sr-BGNPs, and PLG-*g*-TA/VEGF/Sr-BGNPs hydrogels were prepared in 24-well plates with 3 replicate wells for each group. RBMSCs were seeded on the hydrogels at a density of 2×10^4 cells/well and placed in 24-well plates. After incubation for 48 h, the hydrogels were washed with PBS and fixed with 4% paraformaldehyde for 30 min. Then, the fixed samples were washed with PBS, dehydrated in a series of graded ethanol (70%, 80%, 90%, 95%, 100%, and 100%), and dried in a lyophilizer for 24 h. Finally, the samples were sputter-coated with gold for SEM observation.

4.9.4 Cell proliferation

The PLG-*g*-TA, PLG-*g*-TA/VEGF, PLG-*g*-TA/Sr-BGNPs, and PLG-*g*-TA/VEGF/Sr-BGNPs hydrogels were immersed in OriCell® rBMSCs osteogenic differentiation medium at a ratio of 1:10 (v/v). The supernatant was collected as D1 after 24 h. Subsequently, fresh medium was added, the supernatant was collected as D3 at 72 h after the first immersion, and so on for D5. The supernatants were filtered through a 0.22 μm filter membrane for use. RBMSCs were plated into a 96-well plate at a density of 4×10^3 cells/well (CCK-8) and a 24-well plate at a density of 1×10^4 cells/well (live/dead staining). Each group was set up with 3 replicate wells and 3 time points (1, 3, and 5 days). The next day, in the blank group, the medium was replaced with the complete medium, and in each hydrogel group, the medium was replaced with D1 from each group. After 24 h, the cells in the 24-well plates were subjected to live/dead staining, and the cells in the 96-well plates were subjected to a CCK-8 assay. Moreover, the cells in the remaining plates were washed with PBS twice, and the medium of each group was replaced with complete medium or D3 from each group. After 72 h, the cells in the 24-well and 96-well plates were subjected to the same protocol for 24 h, and the media of the remaining groups were replaced with complete medium or D5 from each group. Finally, after 5 days, the cells in the 24-well and 96-well plates were tested as previously described.

4.9.5 Scratch assay

The PLG-*g*-TA, PLG-*g*-TA/VEGF, PLG-*g*-TA/Sr-BGNPs, and PLG-*g*-TA/VEGF/Sr-BGNPs hydrogels were immersed in DMEM (1% penicillin–streptomycin) at a ratio of 1:10 (v/v). The supernatant was collected after 48 h and filtered through a 0.22 μm filter membrane. The *in vitro* migration of cells was determined by a scratch assay. Briefly, the bottom of a 6-well plate was marked with three evenly distributed horizontal lines. Cells were plated into 6-

well plates at a density of 1×10^5 cells/well. When the confluence reached approximately 90%, two parallel scratches were made with the tip of a 200 μL pipette perpendicular to the aforementioned horizontal lines. After the cells were washed with PBS three times, 1.5 mL of DMEM (1% FBS, 1% penicillin–streptomycin) was added to the blank group, while 1.5 mL of the extract solution containing 1% FBS was added to the other groups. Cell migration was visualized using an optical microscope, and the width and area of the scratches at 0 h were recorded. The cells were placed in an incubator at 37 °C, and the images were taken 24 and 48 h later. ImageJ software was used to calculate the scratch area at each time point, and three parallel samples were used for each group.

4.9.6 Evaluation of angiogenesis in vitro

A tube formation assay was conducted to evaluate the angiogenic ability of HUVECs *in vitro* by cocultivation with blank, PLG-*g*-TA, PLG-*g*-TA/VEGF, PLG-*g*-TA/Sr-BGNPs, and PLG-*g*-TA/VEGF/Sr-BGNPs hydrogels. Briefly, the medium was changed to serum-free DMEM/F-12 (1:1, 1% penicillin–streptomycin), and the cells were starved for 24 h before the experiment. At the same time, the PLG-*g*-TA, PLG-*g*-TA/VEGF, PLG-*g*-TA/Sr-BGNPs, and PLG-*g*-TA/VEGF/Sr-BGNPs hydrogels were immersed in DMEM/F-12 (1:1) at a ratio of 1:10 (v/v). The supernatant of the medium was collected 24 h later and filtered through a 0.22 μm filter membrane. The 15-well U-Slide angiogenesis plates, desired 10 μL pipette tips, and 1 mL syringes were first precooled in a -80 °C freezer in advance, and the Matrigel was stored in a 4 °C freezer and melted for use.

The precooled plates were flatly placed on an ice box, and 10 μL of Matrigel was added to each well. Three replicate wells were set up for each group. Then, the plate was transferred to a cell incubator for 30 min until the Matrigel solidified. After the HUVECs were starved for 24 h, subjected to trypsin digestion, terminated, centrifuged, and the supernatant was discarded, the blank group was resuspended in serum-free DMEM/F-12, and the remaining groups were resuspended in their respective extract solutions. A 50 μL cell suspension was vertically seeded on Matrigel at a density of 1.5×10^4 cells/well. Then, the plates were incubated in an incubator until a large vascular network was observed to form under an optical microscope. The medium in each well was aspirated, and the wells were washed with PBS. Then, 50 μL of PBS containing calcineurin was added to the wells for cell staining, and the cells were photographed with a fluorescence microscope. The results were analyzed by ImageJ software.

4.9.7 Activity of alkaline phosphatase (ALP)

The PLG-*g*-TA, PLG-*g*-TA/VEGF, PLG-*g*-TA/Sr-BGNPs, the PLG-*g*-TA/VEGF/Sr-BGNPs hydrogels were immersed with osteogenic differentiation medium at a ratio of 1:10

(v/v). The supernatant was collected as D1 after 24 h. Subsequently, fresh medium was added, and the supernatant was collected as D3 at 72 h after the first immersion. The supernatants were filtered through a 0.22 μm filter membrane. RBMSCs were plated into a 24-well plate at a density of 2×10^4 cells/well. When the confluence reached approximately 70%, the medium was aspirated, and in the blank group, osteogenic differentiation medium was added, while in the hydrogel groups, D1, D3, D5, or D7 extract solution was added to the corresponding groups on Days 0, 2, 4, or 6 of induction, respectively. ALP staining was performed on Day 7. The cells were fixed with 4% paraformaldehyde for 30 min and stained by with ALP staining solution prepared following the protocol of the BCIP/NBT alkaline phosphatase chromogenic kit. The staining area was observed using an optical microscope.

4.9.8 Mineralization of rBMSCs

The cells were grouped and treated according to the same protocol described in section "4.9.7 Activity of alkaline phosphatase (ALP)". On Days 1, 3, 5, 7, 9, 11, and 13, the medium was changed for each group. Then, on Day 14, the cells were fixed with 4% paraformaldehyde for 30 min and stained with alizarin red (ARS). The staining area and the mineralized nodules were observed using an optical microscope.

4.9.9 Immunofluorescence staining of osteogenesis-related proteins

The extract solution was prepared according to the same protocol described in section "4.9.7 Activity of alkaline phosphatase (ALP)". RBMSCs were plated into 6-well plates at a density of 9×10^4 cells/well. Cells were grouped and treated according to the same protocol described in section "4.9.8 Mineralization of rBMSCs". On Day 14, the cells in each group were digested, terminated, centrifuged, resuspended, and reinoculated in 15 mm diameter confocal dishes overnight at a density of 1×10^5 cells/well. The next day, the cells were washed with PBS twice and fixed with 4% paraformaldehyde for 30 min. Then, 0.5% Triton X-100 solution was added to permeabilize the membrane for 30 min, and the solution was discarded. Then, the cells were washed with PBS and blocked by 300 μL of goat serum for 30 min. Subsequently, the cells were incubated with 300 μL of primary antibodies against BMP-2 (1:100, ab214821, Abcam, UK), RUNX2 (1:100, 20700-1-AP, Proteintech, USA), and COL I (1:1000, 14695-1-AP, Proteintech, USA) overnight at 4 °C. The next day, the cells were washed with PBS and further incubated with 300 μL of fluorescein isothiocyanate (FITC)-conjugated AffiniPure goat anti-rabbit IgG (H+L) secondary antibody (1:1000) for 2 h in the dark. Then, the cells were incubated with 300 μL of actin-tracker red-rhodamine containing 0.1% Triton X-100 and 1% BSA (1:150, C2207S, Beyotime, China) for 30 min, followed by 5 min of nuclear staining with 4′,6-diamidino-2-phenylindole (DAPI, Beyotime, China). Finally,

immunofluorescence-stained cells were visualized by confocal laser scanning microscopy (CLSM), and the mean fluorescence intensity was calculated by ImageJ software.

4.9.10 Expression of osteogenesis-related genes

The osteogenic performance of the four hydrogels at the gene level was evaluated by calculating the relative mRNA expression of BMP-2, RUNX2, and ALP. The sequences of the primers used are provided in the supporting information **(Table S1)**. Briefly, the cells were grouped and subjected to the same protocol described in section "4.9.7 Activity of alkaline phosphatase (ALP)". On Day 14, total RNA was extracted from the cells with an Eastep® Super Total RNA Extraction Kit according to the manufacturer's protocol. The extracted RNA was reverse transcribed into complementary DNA (cDNA) using a TaKaRa PrimeScript™ RT reagent kit with gDNA Eraser following the manufacturer's protocol. Reverse transcription polymerase chain reaction (RT–PCR) was performed using PowerUp^{TM} SYBR^{TM} Green Master Mix in a CFX96 real-time PCR detection system. β-Actin was used as the reference gene, and the relative mRNA expression of osteogenesis-related genes was calculated by using the $2^{-\Delta\Delta T}$ method. GraphPad Prism 9 software was used to plot the graphs.

4.9.11 Cytoskeleton and TRAP staining of osteoclasts

RAW264.7 cells can form mature osteoclasts (nuclei number ≥ 3) after being induced with 50 ng/mL RANKL for 7 days [3]. RAW264.7 cells were plated into 48-well plates at a density of 4×10^3 cells/well and cultured for 24 h. Subsequently, the medium for the negative blank (RANKL-free) and positive blank groups was replaced with fresh medium, while the medium for the hydrogel groups was replaced with fresh extract medium for the different groups. Meanwhile, RANKL (50 ng/mL) was added to the positive blank group and hydrogel groups. On Days 1, 3, 5, and 6, the medium was changed.

The cytoskeleton of osteoclasts was first incubated with phalloidin to assess the formation of osteoclasts. Briefly, cells were washed with PBS, fixed with 4% paraformaldehyde solution, and permeabilized using 0.5% Triton X-100 on Day 7. Then, the cytoskeleton of the cells was incubated with phalloidin, and the cell nuclei were stained with DAPI. Finally, fluorescence information was acquired by laser confocal microscopy. TRAP staining was further performed to characterize the formation of osteoblasts. Briefly, cells were washed with PBS, fixed with 4% paraformaldehyde solution, and stained with TRAP staining solution prepared according to the manufacturer's protocol. Finally, the staining information was observed under an optical microscope, and the positive areas were quantitatively analyzed using Image-Pro Plus 6.0 software.

4.9.12 Toluidine blue staining of bovine bone slices

Bovine bone slices soaked in 75% alcohol and subjected to UV irradiation were added to a 96-well plate. RAW264.7 cells were plated onto the surfaces of bovine bone slices at a density of 2×10^3 cells/well and cultured for 24 h. The cells were grouped and subjected to the same protocol described in section "4.9.11 Cytoskeleton and trap staining of osteoclasts". On Days 1, 3, 5, 7, 9, and 10, the medium was changed for each group. Then, on Day 11, the bovine bone slices were washed with 0.25 mol/L NH_3H_2O under ultrasonic waves and dehydrated using gradient alcohols (70%, 80%, 90%, 95%, 100%, and 100%) for 5 min each concentration. Subsequently, 1% toluidine blue staining solution was added. Fifteen minutes later, the bovine bone slices were washed with deionized water. Finally, the staining information was observed under an optical microscope.

4.9.13 Expression of osteoclastic marker genes

Four selected marker genes involved in osteoclastogenesis, Trap (ACP5), NFATc1, c-Fos, and MMP9, were selected to further verify the inhibitory effects of the composite hydrogel on osteoclastic differentiation. The sequences of the primers used are provided in the supporting information **(Table S1)**. Briefly, the cells were grouped and subjected to the same protocol described in section "4.9.11 Cytoskeleton and trap staining of osteoclasts". On Day 7, total RNA was extracted from the cells via the same protocol described in section "4.9.10 Expression of osteogenesis-related genes".

4.10 *In vivo* experiments

4.10.1 Surgical procedure for in vivo animal studies

A rat cranial bone defect model was used to detect bone formation *in vivo* after hydrogel treatment. Fifteen male SD rats (200 to 250 g, 8 weeks old) were provided by the Laboratory Animal Center of West China Hospital of Sichuan University and were cultivated in a standardized animal room at a constant temperature of 22 ℃. They were divided into five groups (blank, PLG-*g*-TA, PLG-*g*-TA/VEGF, PLG-*g*-TA/Sr-BGNPs, and PLG-*g*-TA/VEGF/Sr-BGNPs).

After adaptation for one week, the rats were anesthetized with 4% isoflurane through a small-animal anesthesia machine [4]. The same person established a rat cranial bone defect model based on Patrick P's method **(Figure S20)** [5]. Briefly, the cranium of each rat was shaved and disinfected after anesthesia. An incision of approximately 2 cm down to the periosteum over the scalp from the nasal bone to just caudal to the middle sagittal crest or bregma was made and the subcutaneous tissue was bluntly dissected. A bone defect with a diameter of 5 mm was created on the right side of the parietal bone using trephine drilling at

2000 rpm with sterile normal saline dropwise at approximately 1 drop every 2 s to irrigate the trephine and calvarium to prevent thermal injury. The blank group had cranial bone defects that were not subjected to treatment, while the other groups were treated with different hydrogels with a diameter of approximately 5 mm and a depth of 1 mm. The periosteum was closed using 4-0 Monocryl sutures, and the skin incision was closed using 4-0 silk sutures. The nails of the bilateral forefeet of the rats were clipped to avoid scratching the postoperative sutures, and the rats were placed in rearing cages padded with thermostatic heating blankets at the bottom when they showed obvious signs of activity. Three postoperative doses of buprenorphine for analgesia at 12, 24, and 36 h.

4.10.2 Micro-CT scanning

At the 8th week after surgery, all the rats were euthanized, and the calvarium samples were collected and fixed with formalin for 2 days. Then, micro-CT was used to scan the samples with an X-ray tube set at 70 kV and 100 μA. Skyscan software was used for image reconstruction and data analysis to calculate the bone regeneration at the cranial bone defect site of rats in each group, including bone volume/tissue volume (BV/TV), bone mineral density (BMD), trabecular number (Tb.N), trabecular separation (Tb.Sp), and trabecular thickness (Tb.Th).

4.10.3 Histological staining

After micro-CT scanning, the calvarium samples were decalcified in 10% ethylenediaminetetraacetic acid (EDTA) (pH=7.2) at a ratio of 1:10 (v/v) for 1 month. Then, the samples were put into an automatic dehydrator for dehydration, transparency, and wax dipping. After that, the samples were embedded in paraffin, cut into 4 μm thick slices, and then put into a temperature oven at 65 °C for baking and dewaxing for 60 min. The slices were soaked in xylene I and II for 10 min and then placed in graded alcohol (100%, 100%, 95%, 85%, 75%) for 5 min at each concentration. The slices were immersed in deionized water for 3 min, followed by subsequent experiments.

The histological regeneration of bone defects after treatment with the composite hydrogel was determined by H&E and Masson's trichrome staining. In addition, at the 8th week after surgery, major organs (heart, liver, spleen, lung, and kidney) were also observed by H&E staining to assess the biocompatibility of the hydrogel *in vivo*.

4.10.4 TRAP staining

Trap staining was further performed to characterize the formation of osteoblasts as described in “4.9.11 Cytoskeleton and TRAP staining of osteoclasts”.

4.10.5 Immunofluorescence staining

The expression of osteogenesis-related proteins, such as BMP-2 and RUNX2, was demonstrated by the tyramide signal amplification (TSA) staining strategy following the manufacturer's protocol. Briefly, slices were first processed for antigen repair with EDTA antigen repair solution (pH=9) and antigen closure with antigen sealing solution and then incubated with 100 μL of primary antibodies against BMP-2 (1:100, ab214821, Abcam) for 40 min at 37 °C. Then, after washing with PBS twice and soaking in wash buffer for 1 min, the slices were incubated with Opal Polymer HRP-conjugated anti-mouse secondary antibody for 10 min at 37 °C. After washing with PBS twice, Opal 650 was added for coloration at room temperature for 10 min. Then, the slices were processed for antigen repair and closed again and then incubated with 100 μL of primary antibodies against RUNX2 (1:200, ab236639, Abcam) for 40 min at 37 °C. Then, after washing with PBS twice and soaking in wash buffer for 1 min, the slices were incubated with Opal Polymer HRP-conjugated anti-mouse secondary antibody for 10 min at 37 °C. After washing with PBS twice, Opal 520 was added for coloration at room temperature for 10 min, followed by 5 min of nuclear staining with DAPI. Finally, immunofluorescence-stained slices were visualized by CLSM, and images were analyzed by Qupath 0.3.2 software.

The expression of angiogenesis-related proteins, such as platelet endothelial cell adhesion molecule-1 (CD31), was demonstrated by the TSA staining strategy. Briefly, slices were processed for antigen repair and closed and then incubated with 100 μL of primary antibodies against CD31 (1:100, ab182981, Abcam) for 40 min at 37 °C. After washing with PBS twice and soaking in wash buffer for 1 min, the slices were incubated with Opal Polymer HRP-conjugated anti-mouse secondary antibody for 10 min at 37 °C. After washing with PBS twice, Opal 650 was added for coloration at room temperature for 10 min, followed by 5 min of nuclear staining with DAPI. Finally, immunofluorescence-stained slices were visualized by CLSM, and images were analyzed by Qupath 0.3.2 software.

4.11 Statistical analysis

The data were presented as the mean ± standard deviation (SD). The sample size of biological replicates (n) was given in the corresponding figure legends. One-way analysis of variance (ANOVA) was used to test whether differences between multiple groups were statistically significant. For the real-time PCR results, final normalization was achieved by calculating the ratio of the measured data to the reference data, followed by statistical analysis, while the remaining data were statistically analyzed directly from the measurement results. A p value less than 0.05 was considered to indicate a statistically significant difference. The data

were processed and analyzed with Excel 2019 (Microsoft Corp., Redmond, WA, USA), SPSS 22 (SPSS, Inc., Chicago, IL, USA), and GraphPad Prism 9 (GraphPad Software, San Diego, CA, USA).

References

[1] Z. Li, T. Qu, C. Ding, C. Ma, H. Sun, S. Li, X. Liu, *Acta Biomater* **2015**, *13*, 88, https://doi.org/10.1016/j.actbio.2014.11.002.

[2] O. Tsigkou, S. Labbaf, M. M. Stevens, A. E. Porter, J. R. Jones, *Advanced healthcare materials* **2014**, *3* (1), 115, https://doi.org/10.1002/adhm.201300126.

[3] G. Hong, Z. Chen, X. Han, L. Zhou, F. Pang, R. Wu, Y. Shen, X. He, Z. Hong, Z. Li, W. He, Q. Wei, *Clin Transl Med* **2021**, *11* (5), e392, https://doi.org/10.1002/ctm2.392.

[4] X. Li, B. Ma, J. Li, L. Shang, H. Liu, S. Ge, *Acta Biomater* **2020**, *101*, 554, https://doi.org/10.1016/j.actbio.2019.10.044.

[5] P. P. Spicer, J. D. Kretlow, S. Young, J. A. Jansen, F. K. Kasper, A. G. Mikos, *Nat Protoc* **2012**, *7* (10), 1918, https://doi.org/10.1038/nprot.2012.113.

Supplementary Figures 1 to 22 and Table 1S

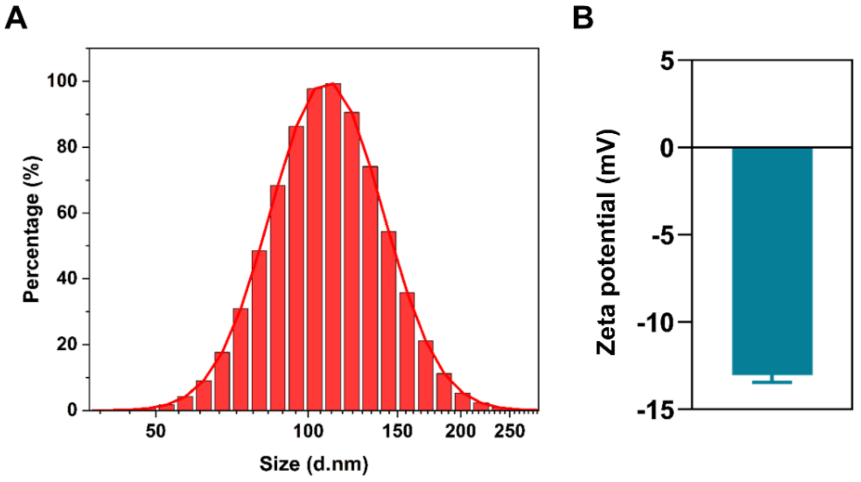

Figure S1. The characteristics of Sr-BGNPs. (A) The particle size distribution measured by dynamic light scattering; (B) the zeta potential of Sr-BGNPs (n = 3 per group).

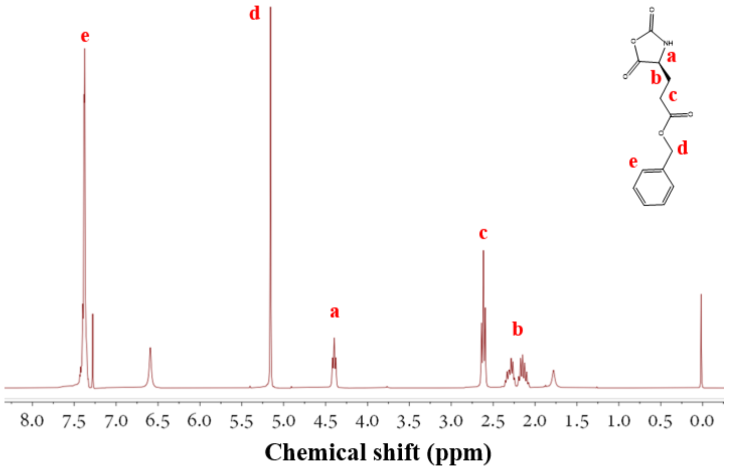

Figure S2. ^{1}H NMR spectrum of the BLG-NCA copolymer.

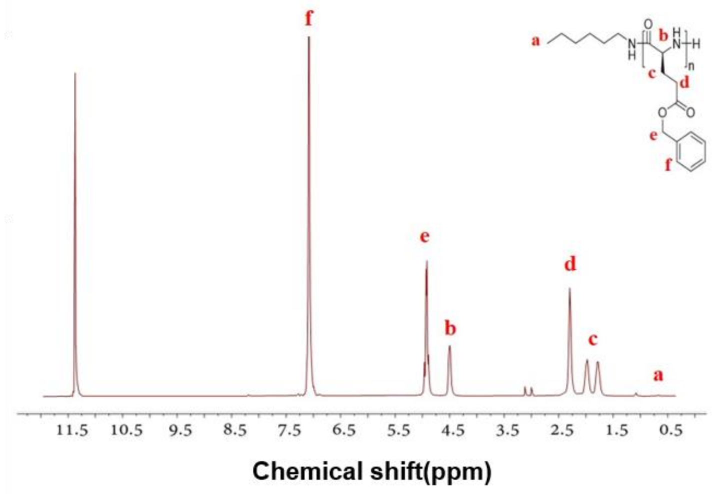

Figure S3. ^{1}H NMR spectrum of the PBLG copolymer.

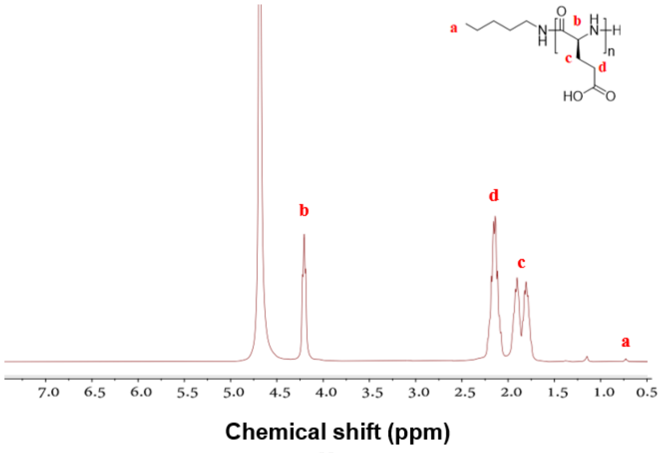

Figure S4. ^{1}H NMR spectrum of the PLG copolymer.

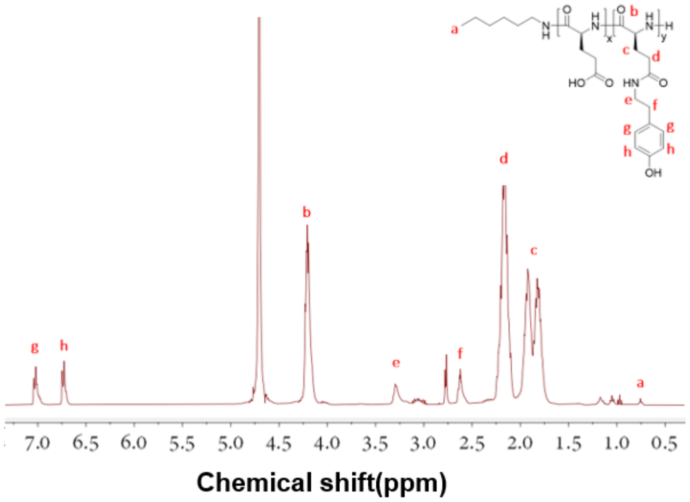

Figure S5. ^{1}H NMR spectrum of the PLG-*g*-TA copolymer.

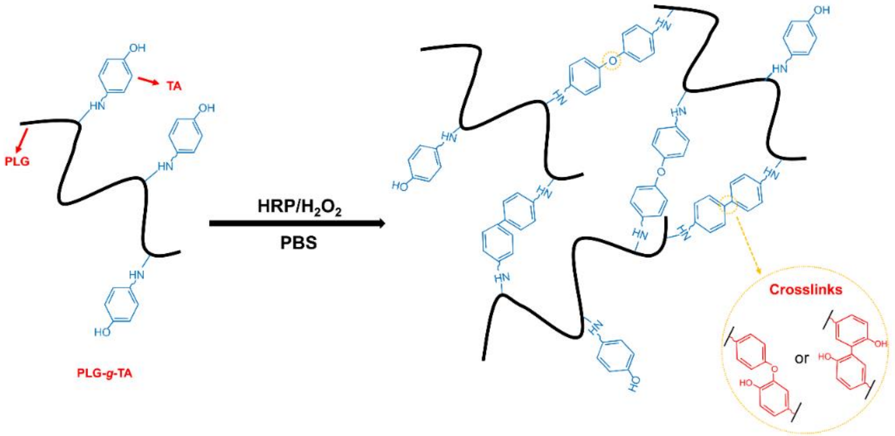

Figure S6. Schematic illustration of the PLG-*g*-TA hydrogels. The free radicals generated by the action of H_2O_2 and HRP act on the phenol groups of PLG-*g*-TA, which can form new free radicals at the position adjacent to the phenol hydroxyl group or hydroxyl group. Then, the neighboring free radicals combine with neighboring free radicals to form a C-C bond or hydroxyl radical combine with neighboring free radicals to form a C-O bond, resulting in intermolecular cross-linking.

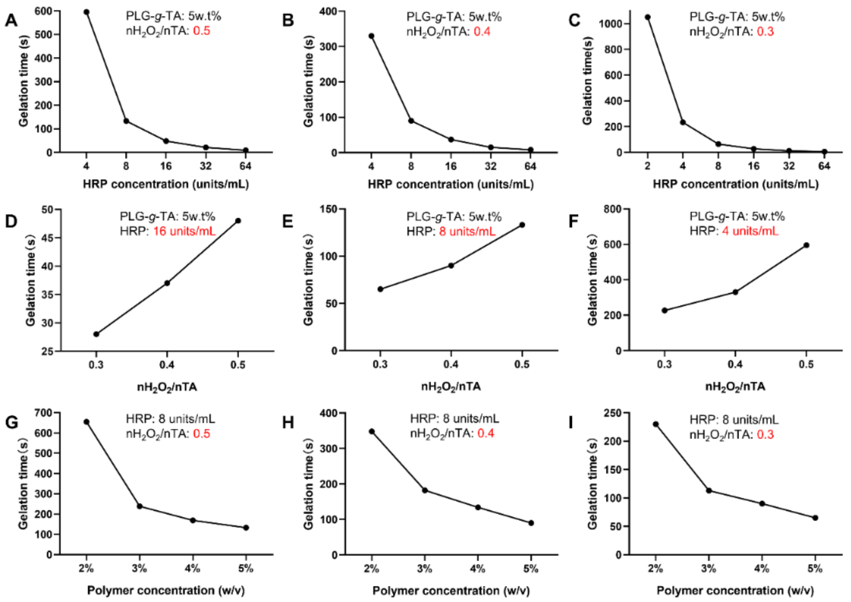

Figure S7. The gelation time of the PLG-*g*-TA hydrogel with different concentrations of H_2O_2, HRP, and the PLG-*g*-TA copolymer.

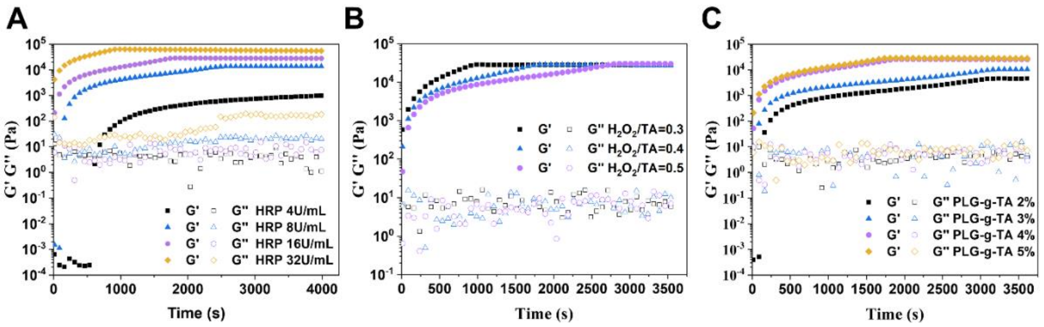

Figure S8. The storage modulus (G') and loss modulus (G") during the gelation process of the PLG-*g*-TA hydrogel under different concentrations of HRP (A), H_2O_2 (B), and the PLG-*g*-TA copolymer (C).

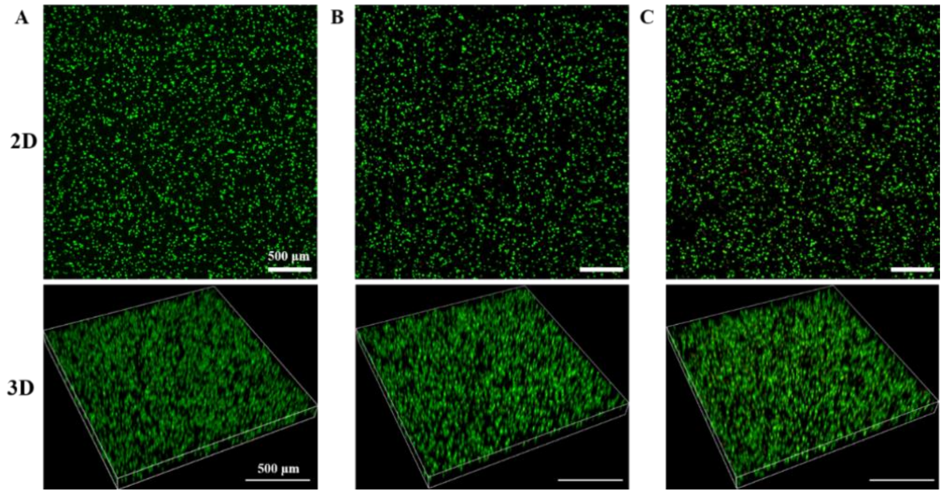

Figure S9. Live/dead staining of rBMSCs after 24 h of coculture in the PLG-*g*-TA hydrogel. All hydrogel precursor polymers had a final concentration of 5% (w/v) and an HRP concentration of 8 units/mL. (A) $nH_2O_2/nTA=0.3$; (B) $nH_2O_2/nTA=0.4$; (C) $nH_2O_2/nTA=0.5$ (scale bar = 500 µm).

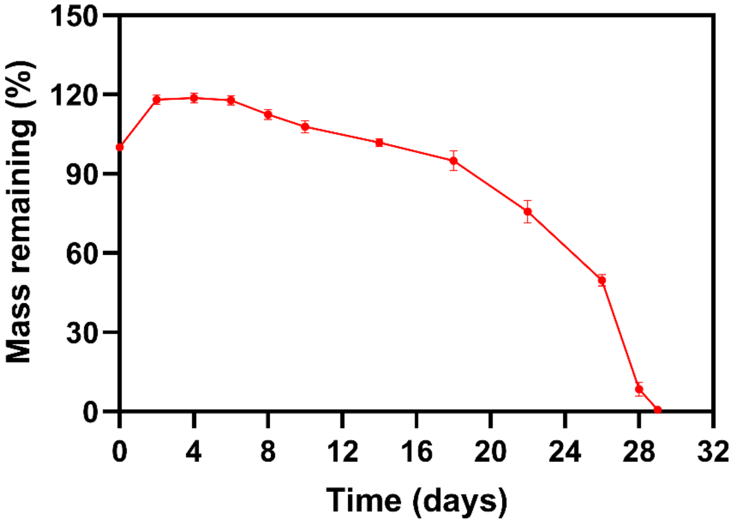

Figure S10. *In vitro* degradation curves of the PLG-*g*-TA hydrogels in PBS supplemented with 2 mg/mL elastase (n = 3 per group).

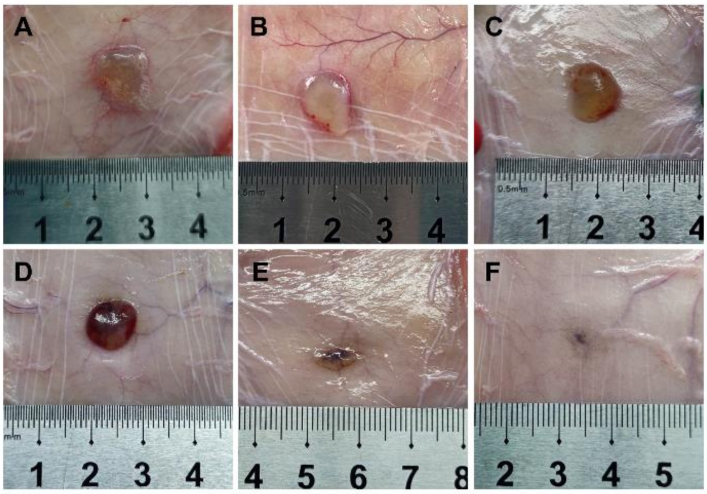

Figure S11. *In vivo* PLG-*g*-TA hydrogel status in the subcutaneous layer of SD rats at different time intervals. Representative images were taken at different time points after the injection. (A) 30 min after injection; (B) 1 week after injection; (C) 2 weeks after injection; (D) 4 weeks after injection; (E) 6 weeks after injection; and (F) 8 weeks after injection.

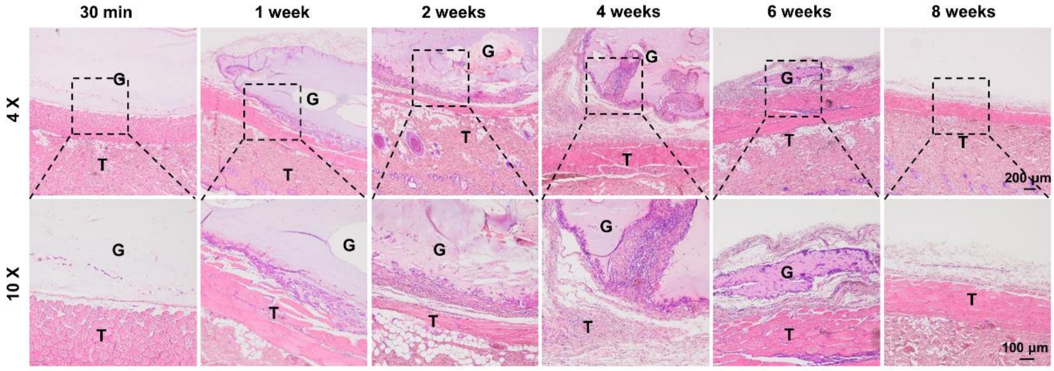

Figure S12. H&E staining images of the skin tissues around the injection sites of the PLG-*g*-TA hydrogel on the backs of the SD rats at different intervals.

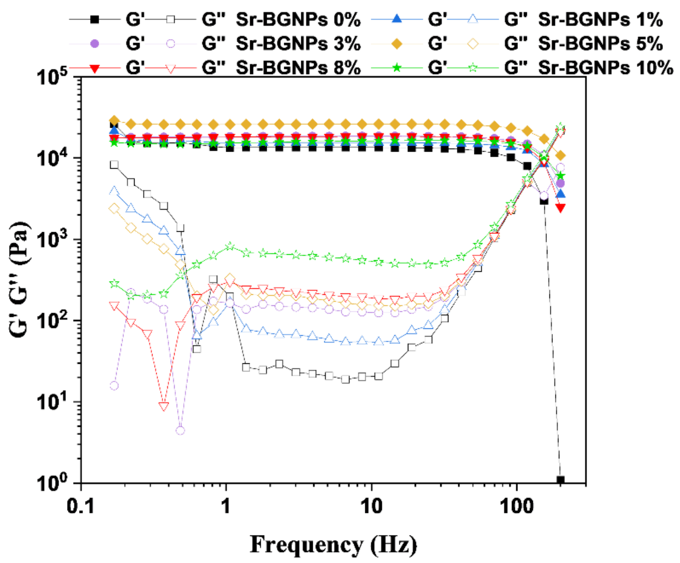

Figure S13. The storage modulus (G') and loss modulus (G") of the PLG-*g*-TA and PLG-*g*-TA/Sr-BGNPs hydrogels were measured via a frequency scan test.

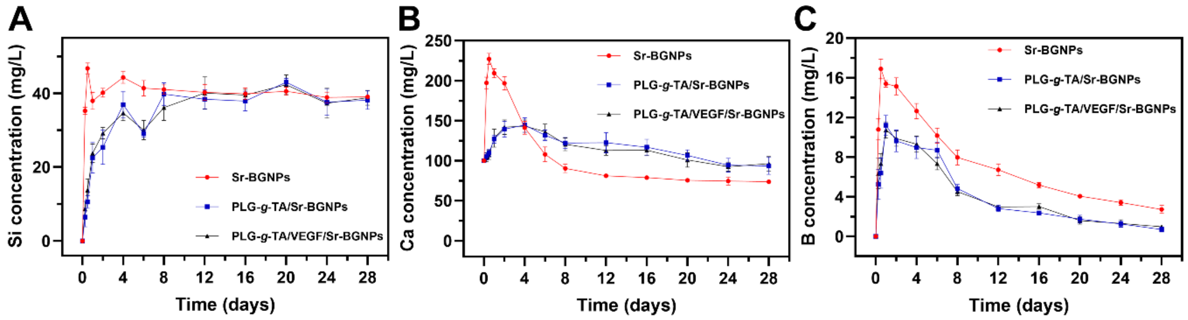

Figure S14. Concentrations of Si (A), Ca (B), and B (C) ions in the medium after 0.25, 0.5, 1, 2, 4, 6, 8, 12, 16, 20, 24, and 28 days of immersion in SBF of Sr-BGNPs and composite hydrogels loaded with Sr-BGNPs (straight lines represent changes in concentration trends, n = 3 per group).

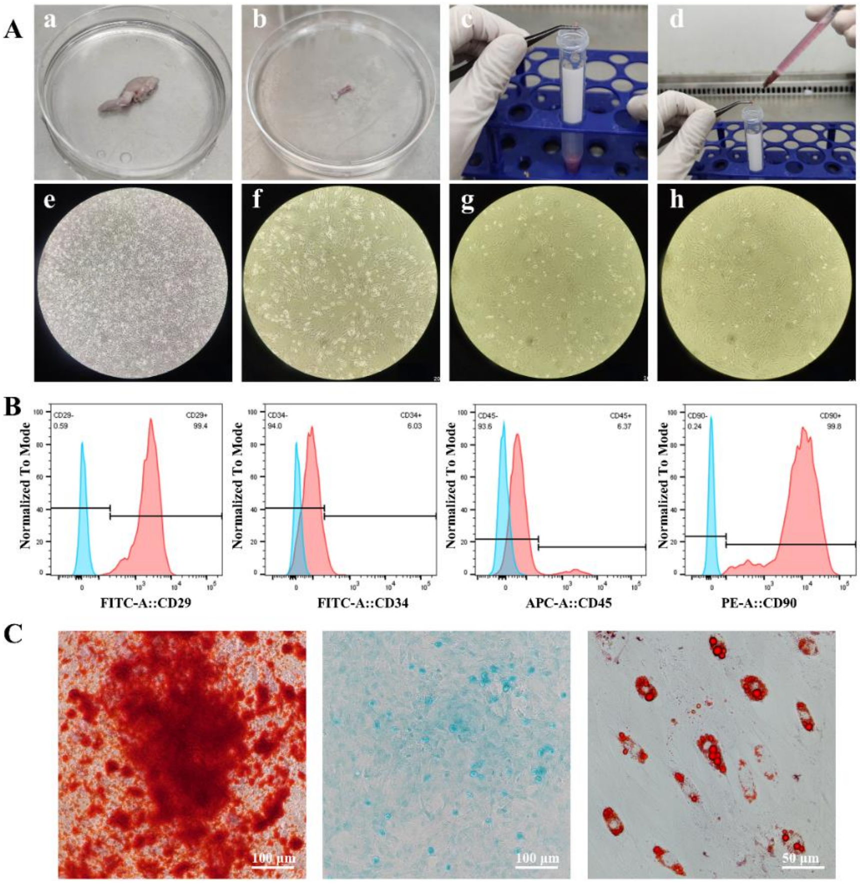

Figure S15. RBMSC extraction and identification. (A) a-d: RBMSCs isolated from the tibial bone marrow of SD rats; e-h: microscopic view of P0 to P3 cell morphology of rBMSCs (10X); (B) the identification of surface markers of rBMSCs performed by flow cytometry: CD29 positive rate 99.4%, CD34 negative rate 94.0%, CD45 negative rate 93.6%, and CD90 positive rate 99.8%, in line with the rBMSC phenotype; (C) the identification of trilineage (osteogenic, chondrogenic, and lipogenic) differentiation ability of rBMSCs.

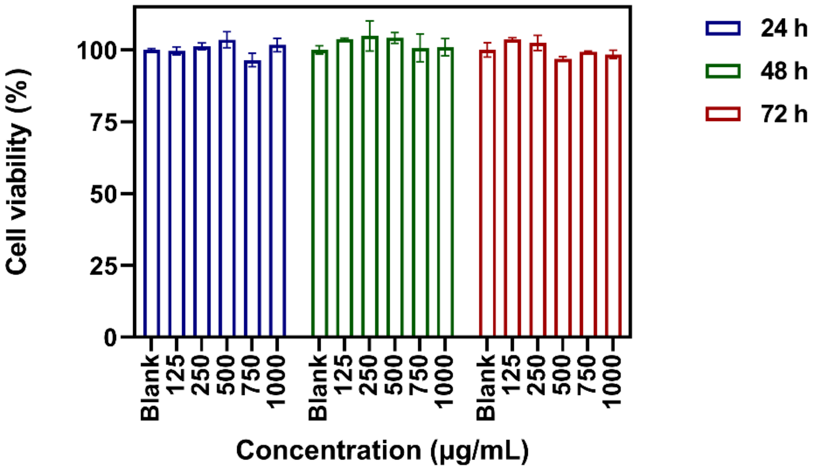

Figure S16. *In vitro* cytocompatibility of the PLG-*g*-TA copolymer using a CCK-8 assay (n = 3 per group).

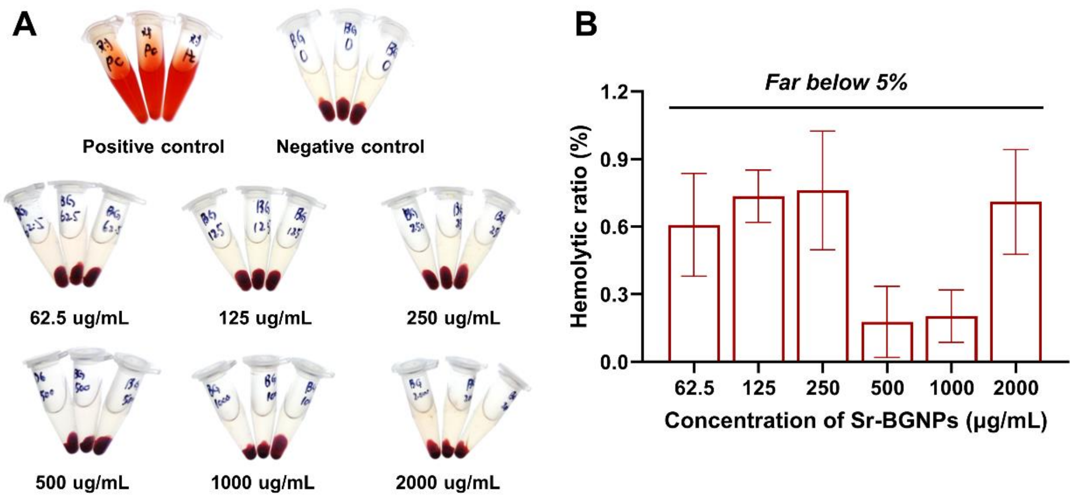

Figure S17. *In vitro* cytocompatibility of Sr-BGNPs. (A) Results of hemolysis experiments with different concentrations of Sr-BGNPs; (B) the hemolytic ratio of different concentrations of Sr-BGNPs (n = 3 per group).

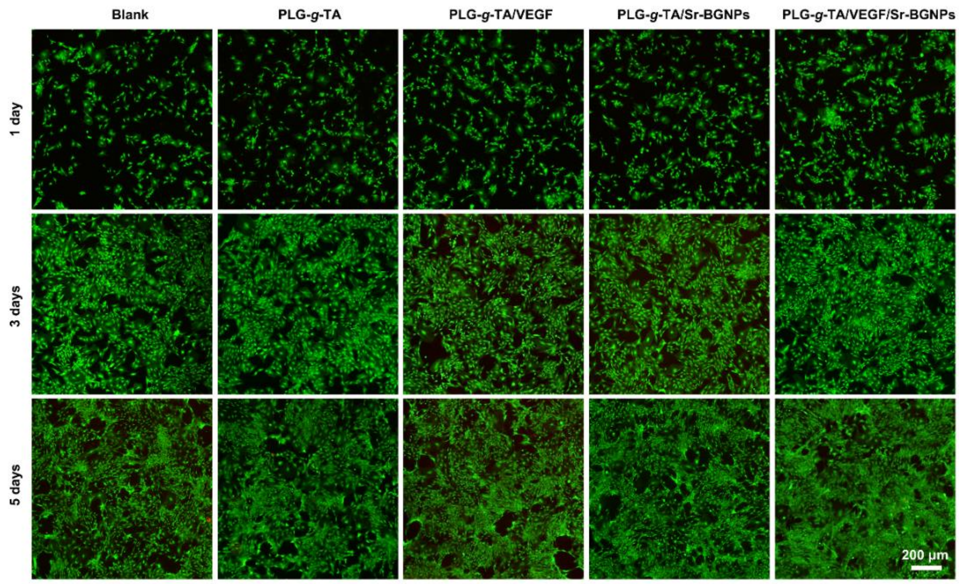

Figure S18. Live/dead staining images of cells cultured for 1, 3, and 5 days in five groups (scale bar = 200 μm).

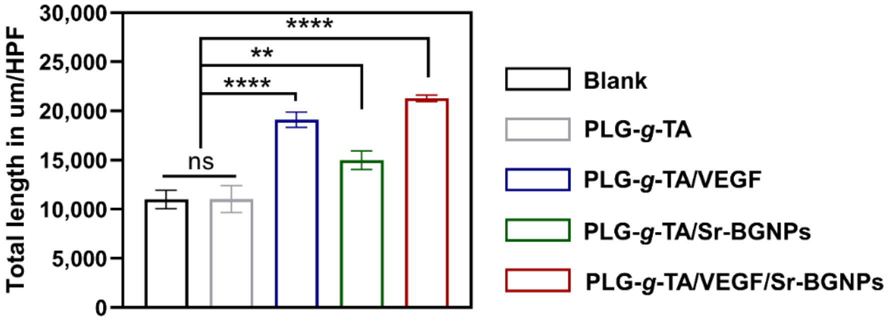

Figure S19. Quantitative analysis of total length per HPF. In this experiment, n = 3 per group. The data are presented as the means ± SDs. Asterisks indicate *p* values; **p* < 0.05, ***p* < 0.01, ****p* < 0.001, *****p* < 0.001, and ns represents no significant difference.

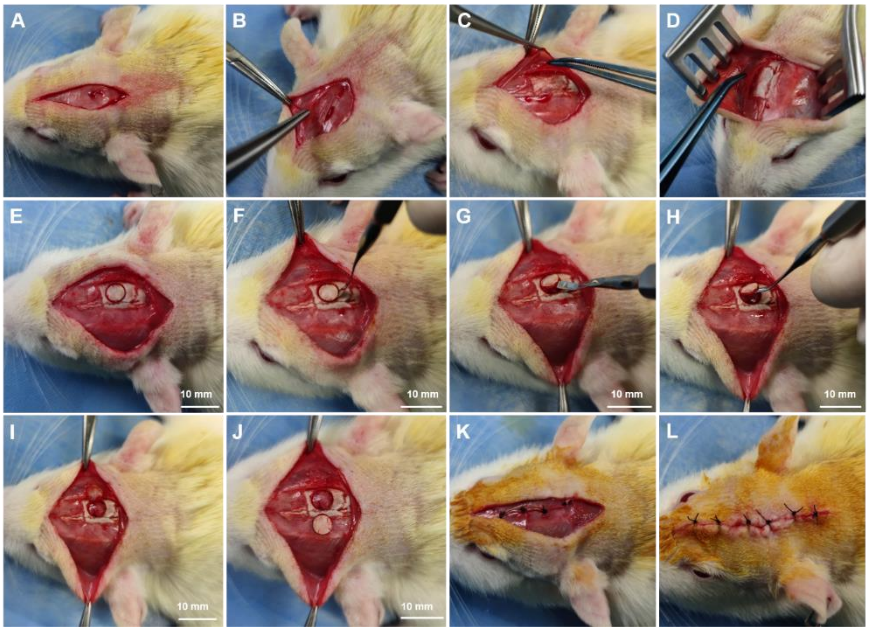

Figure S20. Preparation of the rat calvarial defect model (scale bar = 10 mm).

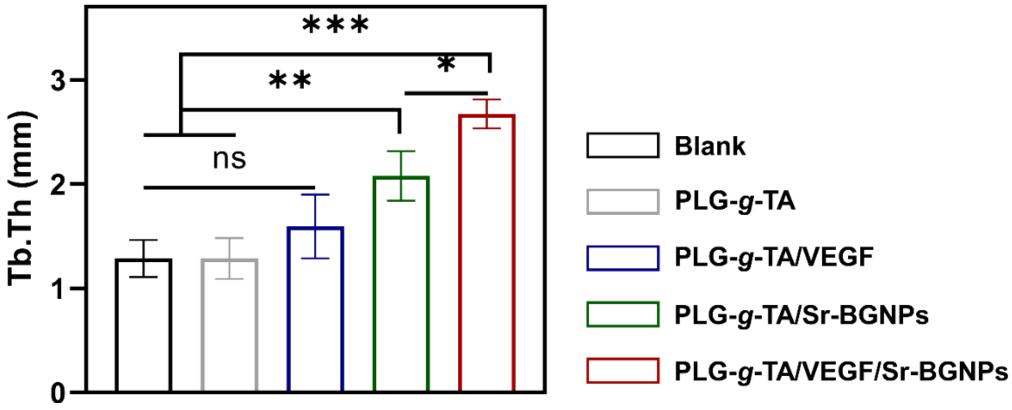

Figure S21. Tb.Th analysis of new bone formation from micro-CT results at 8 weeks after the implantation of hydrogels. The data were analyzed using one-way ANOVA and presented as the means ± SDs. Asterisks indicate *p* values, **p* < 0.05, ***p* < 0.01, ****p* < 0.001, *****p* < 0.0001, and ns represents no significant difference. n = 3 per group.

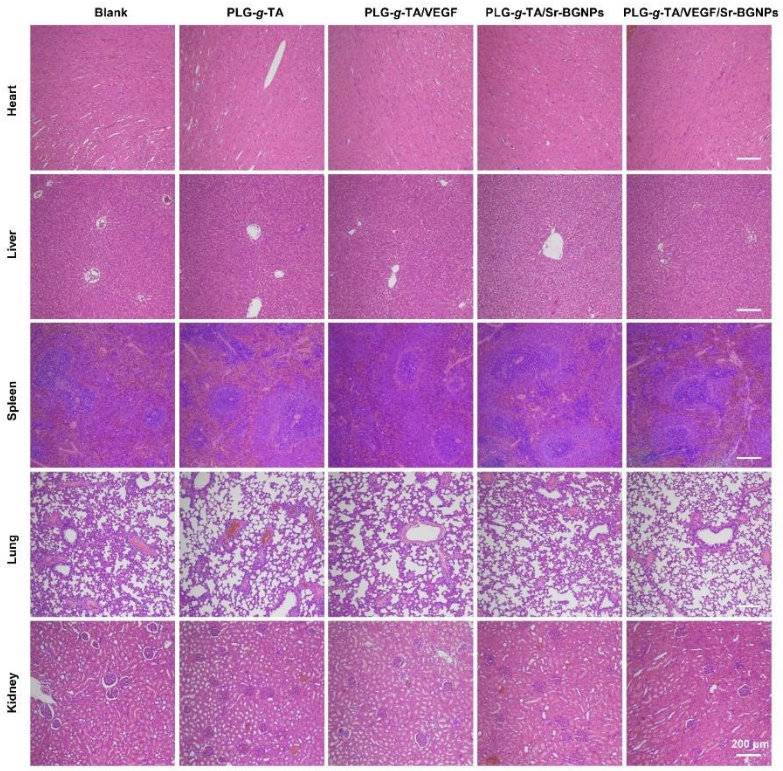

Figure S22. H&E staining of the heart, liver, spleen, lung, and kidney of the rats 8 weeks after implantation of the hydrogels (scale bar = 200 μm).

Table S1. The primer sets used in RT-PCR of this study

Gene name		Primers（5′ to 3′）
β-actin	Forward	AGATCAAGATCATTGCTCCTCCT
	Reverse	ACGCAGCTCAGTAACAGTCC
BMP-2	Forward	CCCCTATATGCTCGACCTGT
	Reverse	CGGGACGTTTTCCCACTCA
RUNX2	Forward	ACAAATCCTCCCCAAGTGGC
	Reverse	GGATGAGGAATGCGCCCTAA
ALP	Forward	TCCTTAGGGCCACCGCT
	Reverse	GGTGTACCCCGAGATCCGTT
Acp5 (Trap)	Forward	TTACTACCGTTTGCGCTTC
	Reverse	CATTTTGGGCTGCTGACT
NFATc1	Forward	CCTTCAGAGAGACCTTGGC
	Reverse	CACAGGAGCTGGGGTTC
c-Fos	Forward	GGGAATGGTGAAGACCGTGT
	Reverse	CCGTTCCCTTCGGATTCTCC
MMP9	Forward	CGACTTTTGTGGTCTTCCCC
	Reverse	CTTCTCTCCCATCATCTGGGC
